# PFKP is a prospective prognostic, diagnostic, immunological and drug sensitivity predictor across pan-cancer

**DOI:** 10.1038/s41598-023-43982-2

**Published:** 2023-10-13

**Authors:** Jian Peng, Pingping Li, Yuan Li, Jichuan Quan, Yanwei Yao, Junfang Duan, Xuemei Liu, Hao Li, Dajiang Yuan, Xiaoru Wang

**Affiliations:** 1 Present Address: Department of Critical Care Medicine, Second Hospital of Shanxi Medical University, Taiyuan, 030001 Shanxi China; 2 Present Address: Comprehensive Liver Cancer Center, The Fifth Medical Center of the PLA General Hospital, Beijing, 100039 China; 3 Present Address: Department of Colorectal Surgery, National Cancer Center/National Clinical Research Center for Cancer/Cancer Hospital, Chinese Academy of Medical Sciences and Peking Union Medical College, Beijing, 100020 China; 4 Present Address: Department of Respiratory and Critical Care Medicine, Second People’s Hospital of Taiyuan, Taiyuan, 030002 Shanxi China

**Keywords:** Cancer genetics, Cancer genomics, Oncogenes, Tumour biomarkers, Tumour immunology

## Abstract

Phosphofructokinase, platelet (PFKP) is a rate-limiting enzyme of glycolysis that plays a decisive role in various human physio-pathological processes. PFKP has been reported to have multiple functions in different cancer types, including lung cancer and breast cancer. However, no systematic pancancer analysis of PFKP has been performed; this type of analysis could elucidate the clinical value of PFKP in terms of diagnosis, prognosis, drug sensitivity, and immunological correlation. Systematic bioinformation analysis of PFKP was performed based on several public datasets, including The Cancer Genome Atlas (TCGA), Cancer Cell Line Encyclopedia (CCLE), Genotype-Tissue Expression Project (GTEx), and Human Protein Atlas (HPA). Prospective carcinogenesis of PFKP across cancers was estimated by expression analysis, effect on patient prognosis, diagnosis significance evaluation, and immunity regulation estimation. Then, pancancer functional enrichment of PFKP was also assessed through its effect on the signaling score and gene expression profile. Finally, upstream expression regulation of PFKP was explored by promoter DNA methylation and transcription factor (TF) prediction. Our analysis revealed that high expression of PFKP was found in most cancer types. Additionally, a high level of PFKP displayed a significant correlation with poor prognosis in patients across cancers. The diagnostic value of PFKP was performed based on its positive correlation with programmed cell death-ligand 1 (PD-L1). We also found an obvious immune-regulating effect of PFKP in most cancer types. PFKP also had a strong negative correlation with several cancer drugs. Finally, ectopic expression of PFKP may depend on DNA methylation and several predicated transcription factors, including the KLF (KLF transcription factor) and Sp (Sp transcription factor) families. This pancancer analysis revealed that a high expression level of PFKP might be a useful biomarker and predictor in most cancer types. Additionally, the performance of PFKP across cancers also suggested its meaningful role in cancer immunity regulation, even in immunotherapy and drug resistance. Overall, PFKP might be explored as an auxiliary monitor for pancancer early prognosis and diagnosis.

## Introduction

Cancer is one of the major causes of human death worldwide. Multiple cancer types have high mortality rates, including lung cancer, stomach cancer, liver cancer, esophageal cancer, colorectal cancer, pancreatic cancer, breast cancer, brain tumor, leukemia, lymphoma, nasopharyngeal cancer, bladder cancer, and cervical cancer.

The International Agency for Research on Cancer reported that nearly 19.3 million new cancer cases and 10 million cancer-related deaths occurred worldwide in 2020^1^. In 2023, it is estimated that there will be 1,958,310 new cancer cases and 609,820 cancer deaths in the United States^2^. In China, the National Cancer Center reported that there were 4,064,000 new cancer cases and 2,414,000 cancer-related deaths in 2016^3^. These data indicate that the high incidence and mortality rates of cancer are still major problems in the word, and there is no more optimism regarding the prevention and control of cancer.

As an ancient bioprocess, glycolysis is the major “motor” and central macronutrient for mammalian organisms to drive cell proliferation, movement, and self-renewal. Specifically, one molecule of glucose splits into two molecules of pyruvate. Then, with oxygen supplementation, pyruvate is oxidized to acetyl-CoA in mitochondria. In the absence of oxygen, lactate is the major reduction product of pyruvate. Finally, ten processes with two ATP molecules were generated^4,5^.

During the malignant progression of cancer, more energy is provided to fulfill its growth advantages, survival, proliferation, and long-term maintenance^6–8^. To satisfy this energy requirement, cancer cells usually reprogram their metabolism process^9–11^. Increasing absorption of glucose and lactate production even in the presence of oxygen and normally functioning mitochondria is a well-known phenomenon, which is defined as the ‘Warburg Effect’^12–14^. The Warburg effect enhances the rate of glucose metabolism 10–100 times the complete oxidation of glucose in mitochondria^12,15^. Therefore, more adenosine triphosphate (ATP) would be synthesized in cancer cells^12,16^. Moreover, intermediate generation and biosynthetic requirements are also enhanced^17,18^. The tumor microenvironment is also altered due to excess lactate secretion, which could enhance tissue architecture disruption and immune cell evasion^19–21^. Finally, cell signal transduction and chromatin modulation could also be altered due to the Warburg effect^22–24^. However, the pancancer role of this process has not been well investigated and concluded.

Several rate-limiting enzymes are involved in mammalian glycolysis, including hexokinase (HK), phosphofructokinase 1 (PFK-1), and pyruvate kinase (PK), whose activity determines the speed and direction of glycolysis^4,25^. PFK-1 is mainly involved in the catalysis of fructose 6-phosphate to fructose 1,6-diphosphate, which is the most important control step of the glycolytic pathway^26,27^. Three subtypes of PFK-1 were observed in mammals: platelet type (PFKP), muscle type (PFKM) and liver type (PFKL)^28^. More recently, studies have shown that PFKP is involved in the initiation and progression of multiple cancer types, such as lung cancer, breast cancer, glioblastoma, T-cell acute lymphoblastic leukemia, and prostate cancer^29–32^. Previous studies have shown that PFKP mainly regulates cancer cell proliferation and metastasis by remodeling glycolysis^29,33,34^. Nevertheless, no additional systematic pancancer analyses of PFKP have been performed, which could help us to predict its diagnostic, prognostic, and immunological significance in different cancer types.

In this paper, we extracted PFKP-related data by traversing diverse cancer-related databases, including The Cancer Genome Atlas (TCGA), Gene Expression Omnibus (GEO), Cancer Cell Line Encyclopedia (CCLE), and human protein atlas (HPA). First, we compared the mRNA expression of PFKP between nontumor and tumor samples across cancers (33 cancer types). Second, prognostic information for PFKP was also displayed to evaluate its clinical significance. Finally, the immune infiltration relationship, correlation with immune-related genes, transcription factor (TF) prediction and functional enrichment were also assessed across cancers. In conclusion, this study mainly summarized the role of PFKP and demonstrated that PFKP may be a candidate proto-oncogene and could be explored as a therapeutic target in most cancer types.

## Methods

### Data collection and expression analysis

Expression data of 31 different normal tissues were downloaded from GTEx (Genotype-Tissue Expression project, https://commonfund.nih.gov/GTEx), which is a resource database and tissue bank for gene expression in human tissues^35,36^. A box diagram of PKFP expression was plotted by the R/Bioconductor package dplyr^37^.

Expression profile data of cancer cell lines were obtained from the Cancer Cell Line Encyclopedia (CCLE, https://sites.broadinstitute.org/ccle). Expression plots of PFKP in cancer cell lines were generated by the R/Bioconductor packages ggpubr and ggplot2^38,39^.

Expression and clinical data across pancancer, including adrenocortical carcinoma (ACC), bladder urothelial carcinoma (BLCA), breast invasive carcinoma (BRCA), cervical squamous cell carcinoma and endocervical adenocarcinoma (CESC), cholangiocarcinoma (CHOL), colon adenocarcinoma (COAD), lymphoid neoplasm diffuse large B-cell lymphoma (DLBC), esophageal carcinoma (ESCA), Glioblastoma multiforme (GBM), head and neck squamous cell carcinoma (HNSC), kidney chromophobe (KICH), kidney renal clear cell carcinoma (KIRC), kidney renal papillary cell carcinoma (KIRP), acute myeloid leukemia (LAML), brain lower grade glioma (LGG), Liver hepatocellular carcinoma (LIHC), lung adenocarcinoma (LUAD), lung squamous cell carcinoma (LUSC), mesothelioma (MESO), ovarian serous cystadenocarcinoma (OV), pancreatic adenocarcinoma (PAAD), pheochromocytoma and paraganglioma (PCPG), prostate adenocarcinoma (PRAD), rectum adenocarcinoma (READ), sarcoma (SARC), skin cutaneous melanoma (SKCM), stomach adenocarcinoma (STAD), testicular germ cell tumor (TGCT), thyroid carcinoma (THCA), thymoma (THYM), uterine corpus endometrial carcinoma (UCEC), uterine carcinosarcoma (UCS), and uveal melanoma (UVM), were obtained from TCGA database with R/Bioconductor package TCGAbiolinks, tidyverse and ggpubr^40–42^. A total of 10,496 samples were collected. Differentially expressed genes (DEGs) across tumor tissues and genes corresponding nontumor tissues in TCGA were analyzed by limma, an R/Bioconductor package for differential expression analysis of RNA-sequencing and microarray studies^43^. The expression data were log_2_ transformed, and statistical significance was estimated by Student's *t* test.

### Protein expression of PKFP across cancers was determined by immunohistochemical staining analysis

The Human Protein Atlas (HPA, https://www.proteinatlas.org/), a human protein atlas for normal and cancer tissues^44–46^, was used to compare the protein level of PFKP across cancers between tumor and nontumor tissues. The PFKP antibody was provided by Sigma‒Aldrich, and the product name was HPA018257 (c = 0.0775 mg/ml), a polyclonal antibody (pAb) generated from rabbit. Cancer types included breast cancer, cervical cancer, liver cancer, lung cancer, pancreatic cancer, prostate cancer, renal cancer, skin cancer, and testis cancer.

### Prognosis analysis of PFKP across cancers

The survival data profile across cancers was downloaded from TCGA by the R/Bioconductor package tidyverse^47^. Overall survival (OS), disease-specific survival (DSS), disease-free interval (DFI), and progression-free interval (PFI) were used to assess the relationship between PFKP level and patient prognosis in ACC, BLCA, BRCA, CESC, CHOL, COAD, COADREAD, HNSC, KICH, LAML, LIHC, LUAD, LUSC, MESO, PAAD, PCPG, READ, SKCM, TGCT, UCS, and UVM. PANCAN survival, containing all tumor patient samples, was also accessed. Log-rank test analysis was performed by the R/Bioconductor packages ggplot2, ggsignif, survminer, and survival^48,49^.

### Diagnosis value analysis of PFKP across cancers

Receiver operating characteristic (ROC) curve analysis and correlation analysis between PFKP and PD-L1 or tumor mutational burden (TMB) were used to evaluate the predictive power of PFKP^50^. ROC curve analysis of PFKP across cancers was performed with the R/Bioconductor package pROC^51^, and area under the curve (AUC) values were calculated to evaluate its diagnostic value (AUC in 0.5–0.6, no diagnostic value; AUC in 0.6–0.75, medium diagnostic value; AUC in 0.75–1.0, perfect diagnostic value)^52^.

Pancancer TMB data were obtained by the R/Bioconductor packages TCGAbiolinks, stringr, and dplyr^37,40,53^. Correlation analysis and plots between PFKP and TMB were performed with R/Bioconductor packages ggstatsplot and ggplot2^54^.

The expression of PFKP and PD-L1 was accessed from the TCGA database with the R/Bioconductor package TCGAbiolinks, and correlation analysis across cancers was performed with ggplot2, ggpubr, and ggpmisc^38,55^.

### Immunity evaluation of PFKP across cancers

Immune scores of pancancer samples were accessed by R/Bioconductor packages utils and estimate^56^. Violin plots were generated with the R/Bioconductor packages ggplot2 and ggpubr.

The immune cell infiltration level of each pancancer sample was assessed through CIBERSORT (https://cibersort.stanford.edu/)^57^, which could calculate the sample immunocyte phenotypes by the gene expression profile. The Pearson correlation coefficient and statistical significance were determined with the R/Bioconductor packages WGCNA^58^, ggpubr and ggpmisc. The correlation heatmap between PFKP and 18 immune cells across cancers was visualized by heatmap, which was similar to the signaling score correlation analysis.

Additionally, pancancer coexpression relationship calculation and plots between PFKP and immune-related genes, including TCR signaling pathway, natural killer cell cytotoxicity, BCR signaling pathway, chemokines, and chemokine receptors, were also used for signaling score correlation analysis.

### Signaling score correlation analysis of PFKP across cancers

The signaling score of each TCGA sample was calculated by R/Bioconductor package progeny, which could accurately estimate pathway activity from gene expression in a wide range of conditions^59^. Then, the correlation of PFKP and different pathway scores was displayed by a heatmap with the R/Bioconductor packages psych, reshape2 and pheatmap^60,61^.

### Drug sensitivity prediction analysis of PFKP across cancers

The drug sensitivity prediction of each TCGA sample was accessed with the R/Bioconductor package oncoPredict, which could predict the response to 198 drugs with screening data^62^. Then, the correlation between PFKP and different drug sensitivity scores was displayed in a scatter plot with the R/Bioconductor package ggplot2. The correlation was estimated with the Pearson correlation analysis.

### Accession numbers of these datasets mentioned above

#### Gene expression (version: 07-21-2019) and survival data (version: 07-19-2019) from TCGA

**Table:** 

Cohort	Gene expression dataset ID	Survival dataset ID
PANCAN	GDC-PANCAN.htseq_fpkm-uq.tsv	GDC-PANCAN.survival-uq.tsv
ACC	TCGA-ACC.htseq_fpkm.tsv	TCGA-ACC.survival.tsv
BLCA	TCGA-BLCA.htseq_fpkm.tsv	TCGA-BLCA.survival.tsv
BRCA	TCGA-BRCA.htseq_fpkm.tsv	TCGA-BRCA.survival.tsv
CESC	TCGA-CESC.htseq_fpkm.tsv	TCGA-CESC.survival.tsv
CHOL	TCGA-CHOL.htseq_fpkm.tsv	TCGA-CHOL.survival.tsv
COAD	TCGA-COAD.htseq_fpkm.tsv	TCGA-COAD.survival.tsv
DLBC	TCGA-DLBC.htseq_fpkm.tsv	TCGA-DLBC.survival.tsv
ESCA	TCGA-ESCA.htseq_fpkm.tsv	TCGA-ESCA.survival.tsv
GBM	TCGA-GBM.htseq_fpkm.tsv	TCGA-GBM.survival.tsv
HNSC	TCGA-HNSC.htseq_fpkm.tsv	TCGA-HNSC.survival.tsv
KICH	TCGA-KICH.htseq_fpkm.tsv	TCGA-KICH.survival.tsv
KIRC	TCGA-KIRC.htseq_fpkm.tsv	TCGA-KIRC.survival.tsv
KIRP	TCGA-KIRP.htseq_fpkm.tsv	TCGA-KIRP.survival.tsv
LAML	TCGA-LAML.htseq_counts.tsv	TCGA-LAML.htseq_counts.tsv
LGG	TCGA-LGG.htseq_fpkm.tsv	TCGA-LGG.survival.tsv
LIHC	TCGA-LIHC.htseq_fpkm.tsv	TCGA-LIHC.survival.tsv
LUAD	TCGA-LUAD.htseq_fpkm.tsv	TCGA-LUAD.survival.tsv
LUSC	TCGA-LUSC.htseq_fpkm.tsv	TCGA-LUSC.survival.tsv
MESO	TCGA-MESO.htseq_fpkm.tsv	TCGA-MESO.survival.tsv
OV	TCGA-OV.htseq_fpkm.tsv	TCGA-OV.survival.tsv
PAAD	TCGA-PAAD.htseq_fpkm.tsv	TCGA-PAAD.survival.tsv
PCPG	TCGA-PCPG.htseq_fpkm.tsv	TCGA-PCPG.survival.tsv
PRAD	TCGA-PRAD.htseq_fpkm.tsv	TCGA-PRAD.survival.tsv
READ	TCGA-READ.htseq_fpkm.tsv	TCGA-READ.survival.tsv
SARC	TCGA-SARC.htseq_fpkm.tsv	TCGA-SARC.survival.tsv
SKCM	TCGA-SKCM.htseq_fpkm.tsv	TCGA-SKCM.survival.tsv
STAD	TCGA-STAD.htseq_fpkm.tsv	TCGA-STAD.survival.tsv
TGCT	TCGA-TGCT.htseq_fpkm.tsv	TCGA-TGCT.survival.tsv
THCA	TCGA-THCA.htseq_fpkm.tsv	TCGA-THCA.survival.tsv
THYM	TCGA-THYM.htseq_fpkm.tsv	TCGA-THYM.survival.tsv
UCEC	TCGA-UCEC.htseq_fpkm.tsv	TCGA-UCEC.survival.tsv
UCS	TCGA-UCS.htseq_fpkm.tsv	TCGA-UCS.survival.tsv
UVM	TCGA-UVM.htseq_fpkm.tsv	TCGA-UVM.survival.tsv

#### Gene expression data from CCLE (cancer cell line encyclopedia)

**Table:** 

Cohort	Dataset ID
mRNA expression	CCLE_Expression_Entrez_2012-09–29.gct

#### GTEx (genotype-tissue expression project)

**Table:** 

Cohort	Dataset ID
mRNA expression	GTEx_Analysis_2017-06-05_v8_RNASeQCv1.1.9_gene_reads.gct.gz

#### HPA (human protein atlas)

**Table:** 

Cohort	Dataset ID
Normal tissue data	normal_tissue.tsv.zip
Pathology data	pathology.tsv.zip
Subcellular location data	subcellular_location.tsv.zip

The immunohistochemical figures were downloaded from HPA by searching the keywords “PFKP”. This website is https://www.proteinatlas.org/ENSG00000067057-PFKP.

### Statistical analysis

All statistical analysis were performed by R language software (version 4.2.2). Statistical significance was estimated with Student's *t* test, if the data obeyed a normal distribution; otherwise, *Mann–Whitney U test* was performed. Pearson correlation analysis was displayed to evaluate the expression correlation between other transcripts, TMB, cell signaling score, drug sensitivity, and immune cells score. All results were considered significant at a *P* value of < 0.05.

#### Ethics approval and consent to participate

No ethical conflicts need to be disclosed in this research.

## Results

### Differential expression of PFKP in normal tissue and cancer cell lines

**Figure 1:**
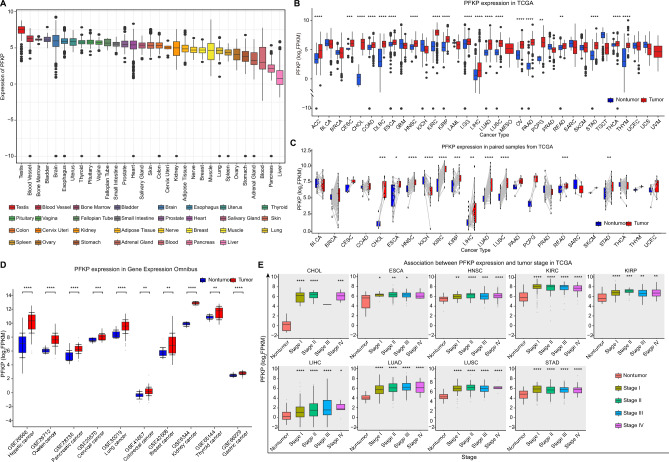
Differential expression of PFKP in normal tissue, cancer cell lines, and tumor samples was analyzed in GTX, CCLE, and TCGA. (**A**) The content of PFKP in normal human tissues is displayed in a box diagram. (**B**) Distinctive expression of PFKP between tumor and nontumor samples was analyzed in TCGA by a box diagram across cancers. (**C**) Expression of PFKP was compared in paired cancer samples across cancers in TCGA. (**D**) Distinction expression of PFKP between tumor and nontumor samples were analyzed in GEO by box diagram across pan-cancer. (**E**) Expression of PFKP in different tumor stages and across cancers is displayed in a box diagram. FPKM, fragments per kilobase million. *p < 0.05, **p < 0.01, ***p < 0.001, ****p < 0.0001. ns, not statistically significant.

To assess the basal expression of PFKP across different human tissues, we analyzed the GTEx data repository, a well-known physiological tissue gene expression profile. Differential expression of PFKP in normal tissues was observed, and PFKP was widely expressed in most normal tissues (Fig. 1A), which suggested its important metabolic function. Our analysis also revealed that the highest expression of PFKP was found in the testis, while the liver possessed the lowest PFKP level (Fig. 1A). Furthermore, we also accessed the CCLE datasets, which cover primary expression data of most cancer cell lines. CCLE data displayed that cell lines from sarcoma had the highest level of PFKP, and the lowest level of PFKP was found in breast neoplasm cell lines. Moreover, most cancer cell lines displayed higher levels of PFKP (Fig. S1A), which was common with normal tissues and indicated an important role across different cancer types.

### Higher expression of PFKP in tumor samples than in nontumor samples across cancers

**Figure 2:**
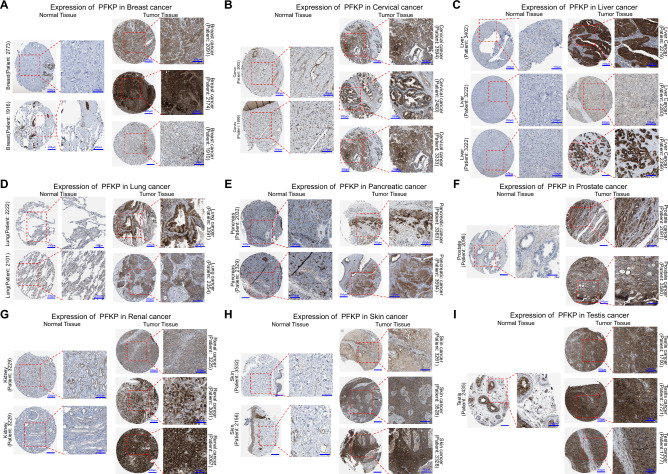
Protein expression of PFKP in tumor samples compared with normal samples across cancers in the HPA. Protein levels of PFKP across tumor and relevant normal samples were analyzed by immunohistochemistry, including breast cancer (**A**), cervical cancer (**B**), liver cancer (**C**), lung cancer (**D**), pancreatic cancer (**E**), prostate cancer (**F**), renal cancer (**G**), skin cancer (**H**), and testis cancer (**I**). Patient id, Patient identifier from HPA. HPA018257, antibody id of PFKP in HPA. Scale bar, 200 μm and 100 μm.

To assess the expression of PFKP in human cancer tissues, we first examined pancancer expression in the TCGA data repository. As Fig. 1B shows, higher expression of PFKP was found in most tumor samples than in nontumor tissues across 60.3% (20/33) of cancer types, including ACC, CESC, CHOL, COAD, DLBC, ESCA, HNSC, KIRC, KIRP, LGG, LIHC, LUAD, LUSC, OV, PAAD, PCPG, READ, STAD, THCA, and THYM. Moreover, we also found that KIRC had the highest level of PFKP, while the lowest cancer type was LIHC (Fig. S1B), which was in accordance with its expression pattern in normal tissues (Fig. 1A). Moreover, paired nontumor and tumor samples from TCGA also displayed a higher PFKP level in tumors (Fig. 1C). Meanwhile, high expression of PFKP in tumor sample was also observed in gene expression omnibus (GEO) data repository across pancancer (Fig. 1D), including hepatic cancer, ovarian cancer, pancreatic cancer, cervical cancer, lung cancer, colorectal cancer, breast cancer, kidney cancer, thyroid cancer, and gastric cancer. Furthermore, to explore the relationship between PFKP and tumorigenesis/progression, the tumor stage relevance of PFKP was determined. As Fig. 1E shows, a higher level of PFKP occurred in earlier stage tumors (Stage I) than in nontumor samples, but no statistical significance was found across different tumor stages, which suggested that PFKP may be mainly involved in tumorigenesis, not progression. Since TCGA data could only reflect the mRNA level of PFKP, we accessed the HPA datasets, which collects most immunohistochemical data of human proteins. In Fig. 2, protein level of PFKP was significantly higher in tumor samples than normal tissues (9 cancer types, including breast cancer, cervical cancer, liver cancer, lung cancer, pancreatic cancer, prostate cancer, renal cancer, skin cancer, and testis cancer). These data suggest that higher expression of PFKP was observed in tumor tissues across cancers.

### Prognostic significance of PFKP across cancers

**Figure 3:**
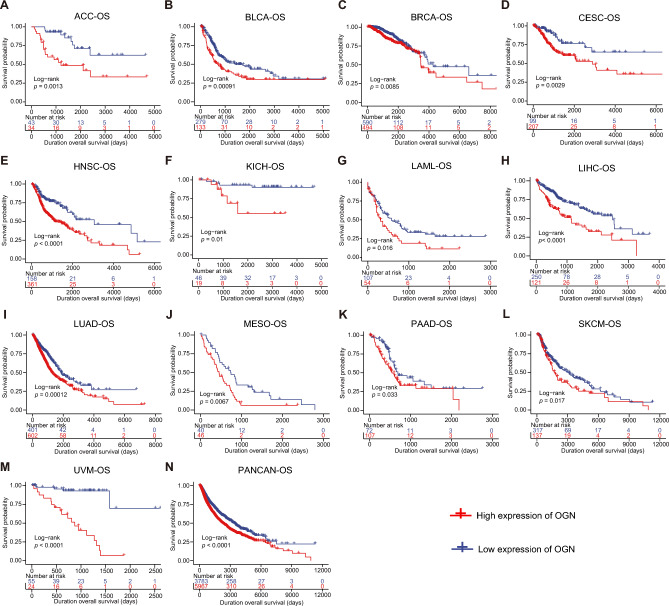
The effect of PFKP expression on overall survival (OS) was assessed across cancers. Overall survival analysis of PFKP was accessed by log-rank test survival analysis in ACC (**A**), BLCA (**B**), BRCA (**C**), CESC (**D**), HNSC (**E**), KICH (**F**), LAML (**G**), LIHC (**H**), LUAD (**I**), MESO (**J**), PAAD (**K**), SKCM (**L**), UVM (**M**), and overall cancer patients (PANCAN) (**N**).

To explore the relevance of PFKP with pancancer prognostic significance, we first analyzed the association between different expression levels of PFKP and patient survival. First, we assessed the overall survival data of PFKP across cancers. As log-rank test overall survival (OS) analysis (Fig. 3) shown, patients with higher expression of PFKP had a worse OS in ACC (*p* = 0.0013), BLCA (*p* = 0.00091), BRCA (*p* = 0.0085), CESC (*p* = 0.0029), HNSC (*p* < 0.0001), KICH (*p* = 0.01), LAML (*p* = 0.016), LIHC (*p* < 0.0001), LUAD (*p* = 0.00012), MESO (*p* = 0.0067), PAAD (*p* = 0.033), SKCM (*p* = 0.017), and UVM (*p* < 0.0001). More importantly, pancancer data also found that a high level of PFKP was related to worse OS (Fig. 3N, PANCAN, *p* < 0.0001). These data suggested that PFKP could be a predictor of survival for patients with cancer.

Second, to assess the role of PFKP in death caused by specific cancers, disease-specific survival (DSS) analysis was performed. According to OS, a high level of PFKP was associated with poor DSS in ACC (*p* = 0.0015), BLCA (*p* = 0.00078), BRCA (*p* = 0.026), CESC (*p* = 4e−04), COAD (*p* = 0.032), COADREAD (*p* = 0.003), HNSC (*p* = 0.0017), KICH (*p* = 0.011), LIHC (*p* = 0.0012), LUAD (*p* = 2e−04), MESO (*p* = 0.034), PAAD (*p* = 0.009), PCPG (*p* = 0.029), READ (*p* = 0.018), SKCM (*p* = 0.012), and UVM (*p* < 0.0001) (Fig.S2A–P). Moreover, in the pancancer range, there was also a positive correlation between a high abundance of PFKP and worse DSS (Fig. S2Q, PANCAN, *p* < 0.0001).

Then, to further clarify the prognostic significance of PFKP, we also screened its relationship with the disease-free interval (DFI) and progression-free interval (PFI). In ACC, BRCA, CHOL, KIRC, LUAD, PAAD, and TGCT (Fig. S3), a significant relationship between high expression of PFKP and worse DFI was observed. Similar results were found between PFKP and PFI in ACC, BLCA, BRCA, CESC, HNSC, KICH, LUAD, LUSC, MESO, PAAD, PCPG, USC, UVM, and PANCAN, as shown in Fig. S4. All these data suggested that PFKP has important clinical significance and may be a better prognostic factor across cancers.

### Diagnostic value of PFKP across cancers

**Figure 4:**
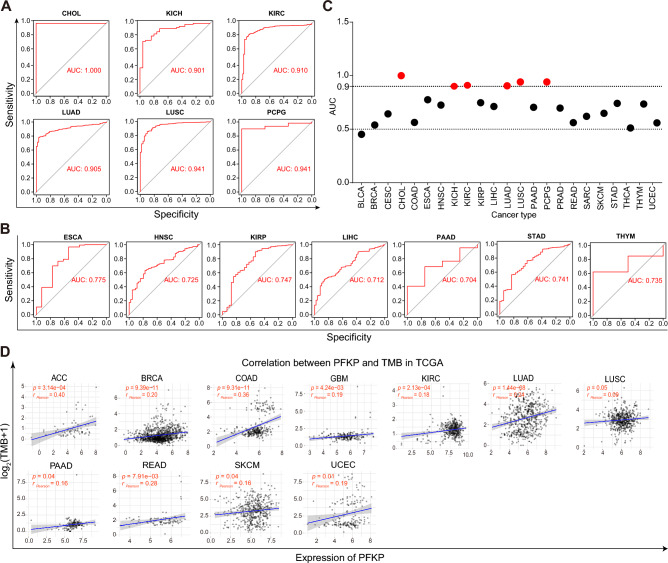
The diagnostic value of PFKP was assessed across cancers. AUC of ROC curves verified the diagnostic significance of PFKP across cancers (**A**, AUC > 0.9; **B**, 0.9 > AUC > 0.7). (**C**) Statistical chart of AUC values for ROC curves. (**D**) Pearson correlation analysis between PFKP and TBM across cancers was performed, and the results are shown in a scatter diagram. TMB, tumor mutation burden; FPKM, fragments per kilobase of exon model per million mapped fragments.

To further explore the clinical significance of PFKP, ROC curves were generated and utilized to estimate the diagnostic accuracy of the signature. As Fig. 4A shows, there was high diagnostic accuracy (AUC: 1.0–0.9) of PFKP in 6 types of cancer. The relative diagnostic accuracy (AUC: 0.9–0.7) of PFKP was observed in ESCA, HNSC, KIRP, LIHC, PAAD, STAD, and THYM (Fig. 4B). The statistical results of the diagnostic accuracy of PFKP across cancers are displayed in Fig. 4C, which indicated a higher correlation between PFKP and diagnostic accuracy.

Additionally, the effect of PFKP on tumor mutational burden (TMB) alteration, which could reflect cancer mutation quantity^63^, was also analyzed across cancers. As shown in Fig. 4D, a positive relationship between them was observed in several types of cancer, suggesting that a high level of PFKP may predict a poor outcome in patients with cancer.

All these data suggested that PFKP had a fine diagnostic value across cancers and could be exploited as a better diagnostic factor.

### Relationship between PFKP expression level and tumor immune cell infiltration

**Figure 5:**
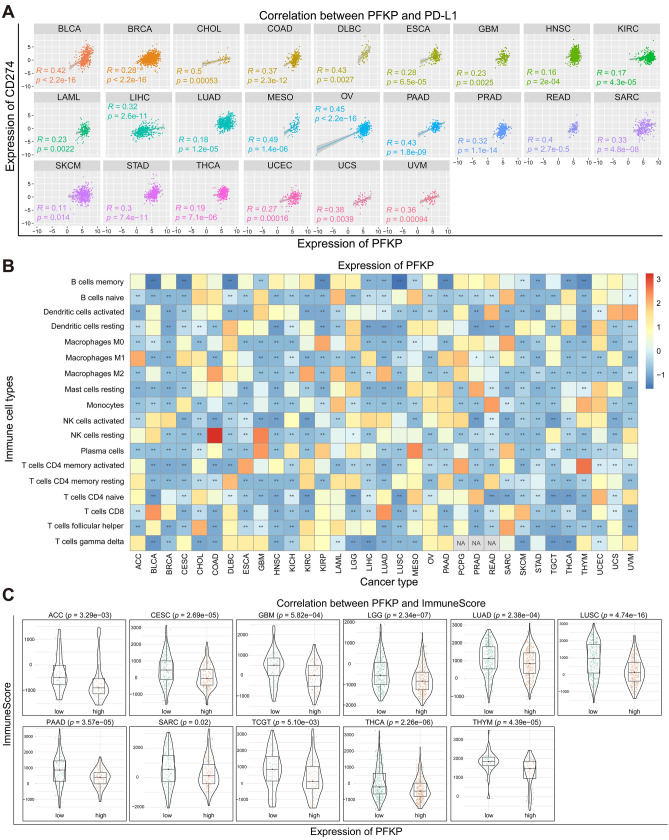
Correlation analysis between PFKP and immune regulation across cancers was performed. (**A**) Pearson correlation analysis between PFKP and PD-L1 (CD274) across cancers was performed by scatter diagram. (**B**) Pearson correlation analysis between PFKP and immune cells across cancers is displayed in a heatmap. (**C**) The effect of PFKP on the immune score across cancers is displayed in violin plots. Low, lower expression group of PFKP; high, higher expression group of PFKP.

Moreover, the relationship between PFKP and programmed death-ligand 1 (PD-L1) in tumors, which is frequently observed in human cancer and has been developed as a biomarker for immune checkpoint inhibitor response^64,65^, is presented in Fig. 5A. Recent data also revealed a positive correlation between PFKP and PD-L1. Specifically, PFKP could promote EGFR activation-induced PD-L1 expression by its nonmetabolic function in human GBM cells^66^. However, no additional clinical data supported this correlation. Our data displayed a significant positive correlation between them in 72.7% (24/33) of cancer types, including BLCA, BRCA, CHOL, COAD, DLBC, ESCA, GBM, HNSC, KIRC, LAML, LIHC, LUAD, MESO, OV, PAAD, PRAD, READ, SARC, SKCM, STAD, THCA, UCEC, UCS, and UVM.

A significant positive correlation between PFKP and PD-L1 suggested that PFKP may be involved in immune cell infiltration across cancers. To solve this question, its relevance to immune cells was assessed. First and foremost, we estimated its correlation with different immune cells in tumor samples from TCGA datasets. As the heatmap (Fig. 5B) shows, a significant negative correlation between them was observed across cancers, indicating lower immune cell infiltration in tumor samples and a worse prognosis for patients. We also assessed the relevance between PFKP and the immune score of tumor samples. Violin plots displayed that higher expression of PFKP was associated with a lower immune score than low expression in 11 types of cancer (Fig. 5C).

**Figure 6:**
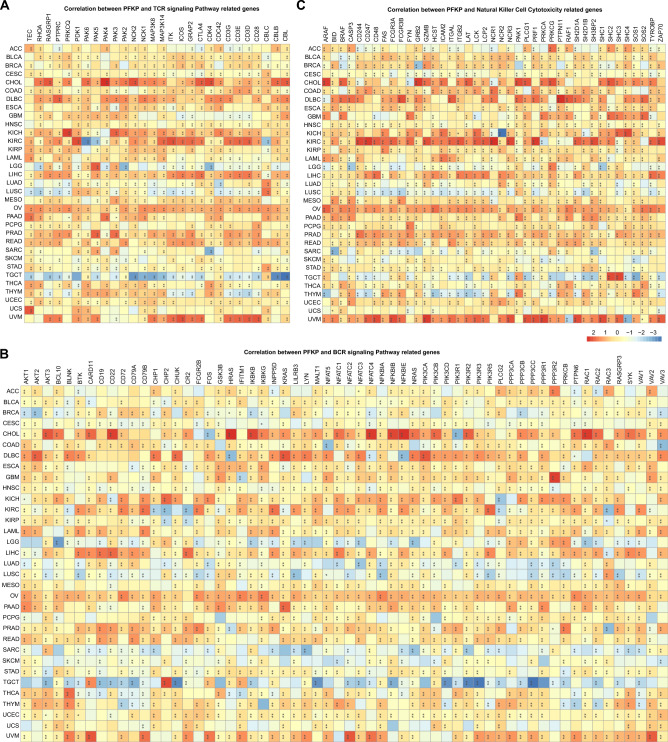
Pearson correlation analysis between PFKP and immune-related genes across cancers is displayed by heatmaps. (**A**) Pearson correlation analysis between PFKP and TCR signaling pathway-related genes is displayed in a heatmap. Correlation between PFKP and 924 TCR signaling pathway related genes were accessed and 60 representative genes are presented. (**B**) Pearson correlation analysis between PFKP and BCR signaling pathway-related genes is displayed in a heatmap. Correlation between PFKP and 1980 BCR signaling pathway related genes were accessed and 60 representative genes are presented. (**C**) Pearson correlation analysis between PFKP and natural killer cell cytotoxicity-related genes is displayed in a heatmap. Correlation between PFKP and 134 natural killer cell cytotoxicity related genes were accessed and 41 representative genes are presented.

To further explore the internal mechanism of FPKP and tumor immune cell infiltration, we hypothesized that PFKP may alter the expression of immune-related genes. For this purpose, we traversed the RNA-seq data from TCGA and found an obvious correlation between PFKP and immune-related genes. Specifically, a generally positive correlation with the TCR signaling pathway which was the core pathway of cellular immune research^67,68^, was presented in Fig. 6A. T-cell development kinases (TECs) were positively correlated with PFKP in 66.67% (22/33) of the types of cancer. Additionally, cytotoxic T-lymphocyte associated protein 4 (CTLA4), which is an important T-cell immune regulation factor and a well-known immune checkpoint target in cancer immunotherapy, was also upregulated in high PFKP cancer samples. Interestingly, most TCR signaling pathway-related genes had a stronger positive correlation with PFKP in CHOL, which suggested that PFKP may have powerful T-cell immune regulation in this cancer type. In contrast, a universally negative correlation between TCR signaling pathway-related genes and PFKP was revealed in TGCTs. All these correlation analyses suggested that PFKP may participate in TCR signaling regulation.

We also found a strong correlation between PFKP and genes related to the BCR signaling pathway (Fig. 6B) or natural killer cell cytotoxicity (Fig. 6C).

Furthermore, because chemokines and chemokine receptors are involved in host defense and immunity^69^, we estimated the effect of PFKP on chemokine- and chemokine receptor-related gene expression. As Fig. S5A shows, a universal positive relevance between these genes was found across cancers. For example, complement C3, the most abundant component of the complement system^70^, had a visible positive correlation with PFKP in 17 types of cancer. Complement C5 had a similar phenomenon as C3. Additionally, a heatmap (Fig. S5A) revealed that chemokines, including the C-X3-C motif chemokine ligand family, semaphorin chemokine family, and slit guidance ligand family, had a significant correlation with PFKP across cancers. A similar result was observed in the correlation between chemokine receptor-related genes and PFKP (Fig. S5B).

In summary, PFKP may play an important role in immune system regulation across cancers as suggested by the strong correlation of PFKP with immune cells, tumor immune cell infiltration, and immune-related genes.

### Functional exploration of PFKP across cancers

**Figure 7:**
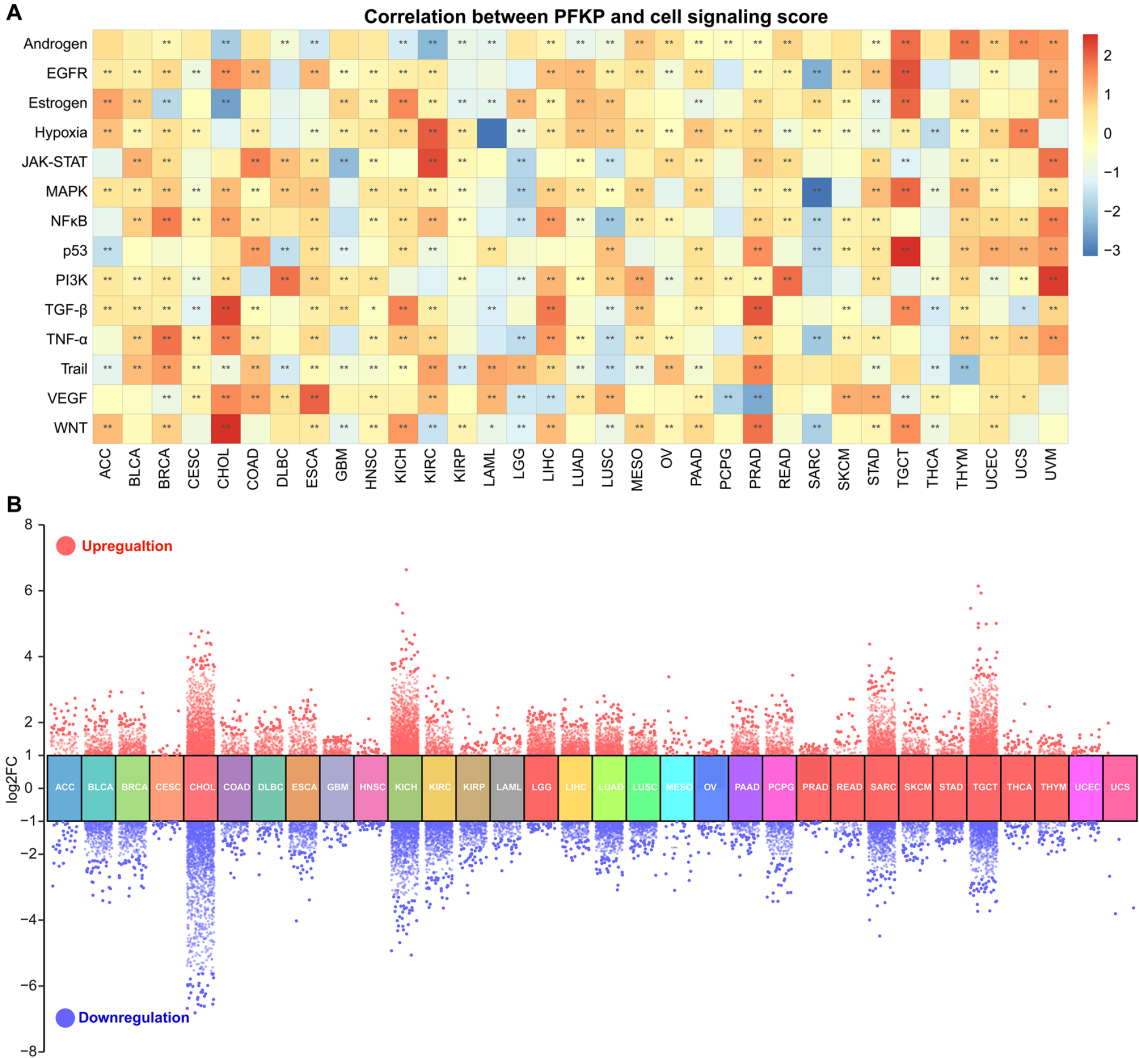
Pancancer functional analysis of PFKP was performed. (**A**) The effect of PFKP on the cell signaling score across cancers is displayed by a Pearson correlation heatmap. (**B**) The effect of PFKP on the gene expression profile across cancers was determined by differentially expressed gene (DEG) analysis and displayed by a grouped volcano plot. FC, fold change.

Previous data reported that highly expressed PFKP is involved in glucose metabolism disturbance in lung cancer^29^. Moreover, stabilization of PFKP in human glioblastoma is involved in tumorigenesis^32^. However, no reports have focused on a general summary of the functions of PFKP across cancers. To resolve this confusion, we evaluated the correlation between PFKP and cell signaling pathways. We found that there was a significant positive correlation between PFKP and WNT, VEGF, TGF-β, PI3K, and hypoxia (Fig. 7A), which are important in cancer development and progression^71–75^. Moreover, a correlation heatmap (Fig. 7A) also revealed that TNF-α, an immune system-related factor^76^, had a strong positive correlation with PFKP across cancers (Fig. 7A, Line 11). This result was also in accordance with previous results.

**Figure 8:**
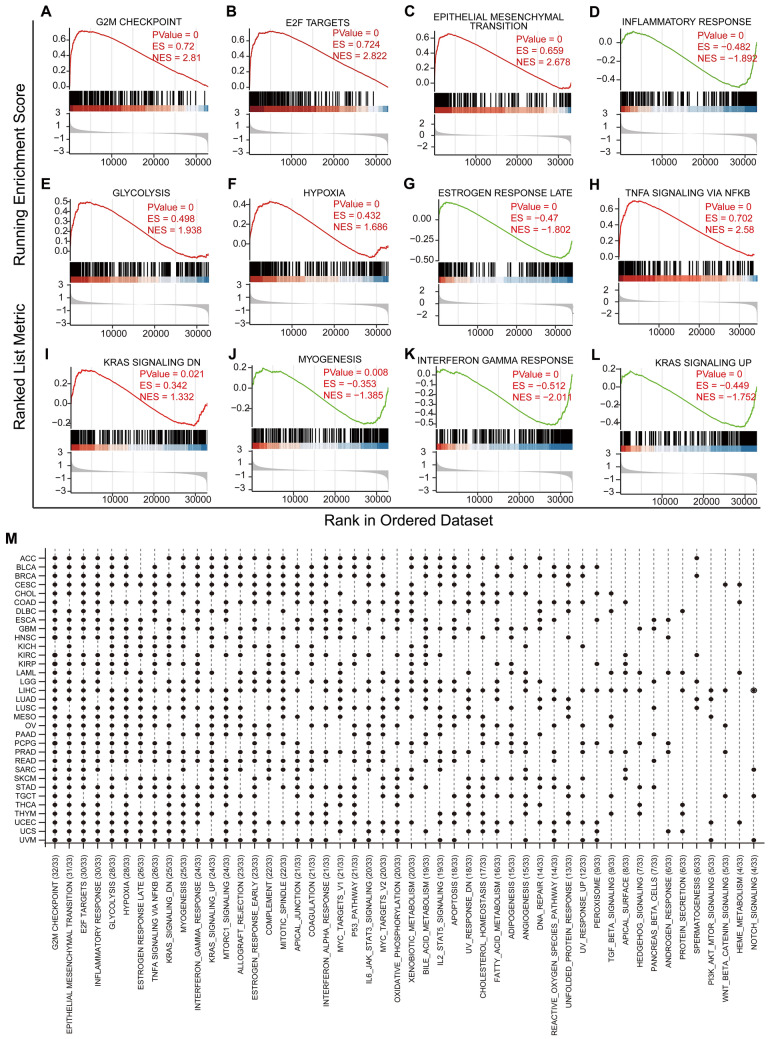
The pancancer function of PFKP was estimated by GSEA (gene set enrichment analysis). The top 12 hallmarks of PFKP-regulated DEGs across cancers are visualized in Panels (**A-L**). (**M**) All hallmarks of PFKP-regulated DEGs across cancers are displayed in a statistical plot. ES, enrichment score.

Furthermore, to clarify and summarize the function of PFKP across cancers, TCGA tumor samples were divided into two groups according to high or low expression of PFKP. Then, we analyzed the DEGs across cancers (Fig. 7B). Additionally, functional enrichment of pancancer DEGs was performed with GSEA (Fig. 8). We found that the top 11 enriched pathways included the G2M checkpoint, E2F targets, epithelial mesenchymal transition (EMT), inflammatory response, glycolysis, hypoxia, late estrogen response, TNF-α signaling via NF-κB, KRAS signaling, myogenesis, and interferon gamma response. These data suggested that PFKP may participate in cancer proliferation by regulating G2/M transition (Fig. 8A) and glycolysis (Fig. 8E), which was consistent with previous research in lung cancer by Shen et al.^29^.

Additionally, GSEA enrichment also revealed that DEGs of PFKP may be the downstream targets of the transcription factor E2F in most cancer types (94%, 31/33) (Fig. 8B,M), which is a significant cell cycle transcription factor^77^. These data suggested that PFKP may regulate cell cycle genes through E2F, but more evidence is needed to support this hypothesis.

Similarly, GSEA also indicated that PFKP may be involved in cancer metastasis by regulating EMT (Fig. 8C), which was also previously reported by Nam Hee Kim^31^.

Moreover, the inflammatory response (Fig. 8D), TNF-α signaling via NF-κB (Fig. 8H), and interferon gamma response (Fig. 8K) enrichment of PFKP DEGs also indicated that PFKP is probably involved in immune modulation to a great extent, which was also consistent with our previous immune modulation results in Figs. 5, 6. More interestingly, GSEA results revealed that DEGs of PFKP had a strong correlation with hypoxia in 85% (28/33) of cancer types (Fig. 8F,M), an important hallmark of cancer^78^. Hypoxia is closely related to glycolysis, which is the initial function of PFKP^79^.

These data suggested that the pancancer function of PFKP not only focused on glycolysis but also included cell cycle regulation by the transcription factor E2F. Furthermore, these data indicate that PFKP may also play an important role in immunoregulation.

### Drug prediction potential of PFKP across cancers.

**Figure 9:**
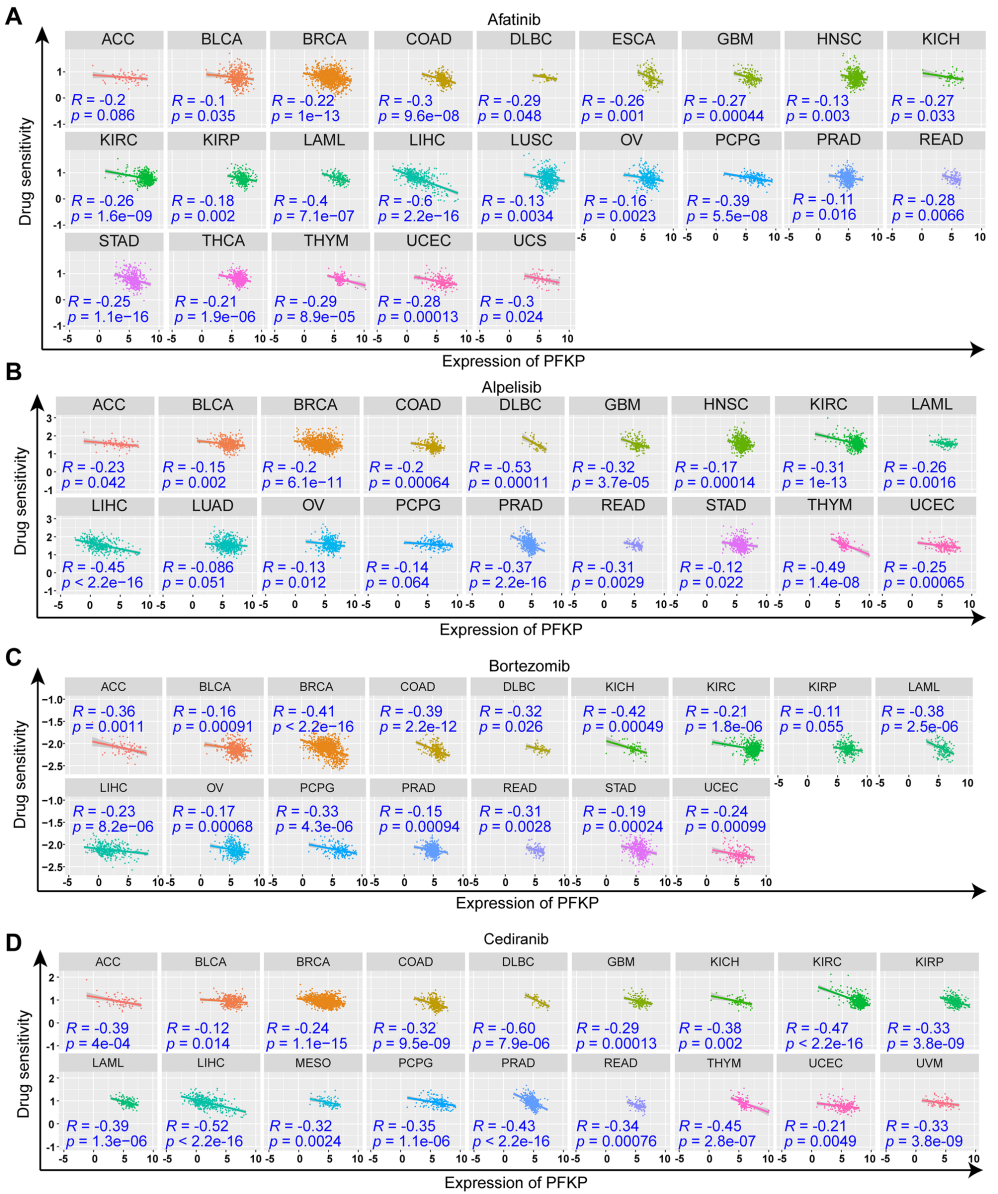
Effect of PFKP on cancer drug sensitivity across cancers. Correlations between PFKP and sensitivity to afatinib (**A**), alpelisib (**B**), bortezomib (**C**), and cediranib (**D**) are displayed in scatter plots. *R*, Pearson correlation coefficient.

To date, few studies have focused on the relationship between PFKP and cancer drugs. To further explore the drug prediction potential of PFKP, we assessed the effect of PFKP on the sensitivity of different cancer drugs. We estimated different cancer drugs for every cancer sample from TCGA datasets. As Figs. 9 and S6 show, there was a significant relationship between PFKP expression and the sensitivity to different cancer drugs (198 cancer drugs were evaluated and 8 representative drugs were selected for presentation). Concretely, a negative correlation between drug sensitivity to afatinib and PFKP expression was observed in 69.7% (23/33) of cancer types (Fig. 9A). Afatinib is a powerful inhibitor of EGFR and human epidermal growth factor receptor 2 (HER2) tyrosine kinase^80–82^, which was consistent with our previous findings in Fig. 7. Moreover, we also noticed this negative correlation with other cancer drugs, including alpelisib (Fig. 9B), bortezomib (Fig. 9C), cediranib (Fig. 9D), osimertinib (Fig. S6A), taselisib (Fig. S6B), ibrutinib (Fig. S6C), and dasatinib (Fig. S6D).

These data suggested that a high level of PFKP could be an indicator of drug resistance to several drugs.

### Expression regulation of PFKP across cancers

**Figure 10:**
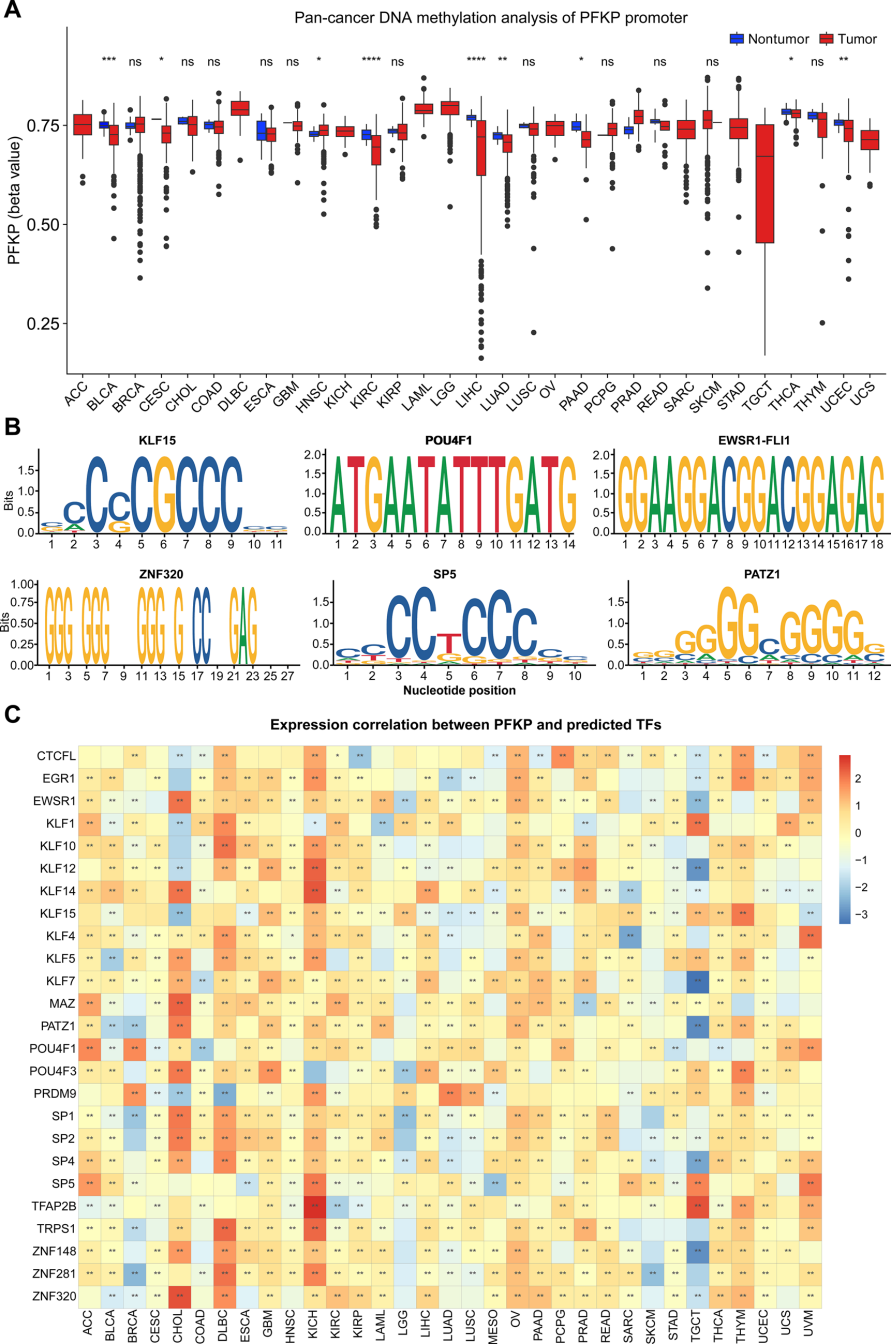
Pancancer regulation of PFKP was analyzed by DNA methylation and transcription factor prediction. (**A**) Promoter DNA methylation comparison of PFKP between tumor and nontumor tissues is displayed in a box diagram. (**B**) Sequence logo plots were used to display several predicted transcription factors’ binding motifs. (**C**) Expression correlation analysis between predicted transcription factors and PFKP is shown by a heatmap.

Finally, we explored the regulation of PFKP expression. First, promoter methylation of *PFKP* between tumor and nontumor tissues was assessed across cancers. The box diagram plotted in Fig. 10A shows that 8 cancer types had a lower methylation level of the *PFKP* promoter in tumor samples versus nontumor samples, which may be responsible for its higher expression level in tumor samples.

Moreover, to further explore the regulation of PFKP expression, we performed transcription factor (TF) prediction with JASPAR (https://jaspar.genereg.net/analysis). As shown in Table S1, several TFs may be recruited to the *PFKP* promoter, including KLF15 (KLF transcription factor 15), POU4F1, EWSR1-FLI1, ZNF320, SP5, and PATZ1. The binding motifs of the top 5 TFs are displayed in Fig. 10B.

Furthermore, to explore the regulation proficiency of these TFs, the expression correlation between PFKP and these predicted TFs was assessed across cancers. As shown in the heatmap (Fig. 10C), there was significant relevance between these factors. Moreover, KICH displayed the most significant positive correlation between them (94%, 31/33) (Fig. 10C, Column 11). We also found that the binding proficiency of the Sp transcription factor family (including SP1, SP2, SP4, and SP5) (Fig. 10C, Rows 17–20) could bind GC/GT-rich promoter elements by its zinc finger structure and play a critical role in tumor growth and metastasis^83^. Our correlation analysis also found that the Kruppel family of transcription factors (KLF) may also be enriched in the promoter of *PFKP*, including KLF1, 4, 5, 7, 10, 12, 14, and 15 (Fig. 10C, Rows 4–11), which was in accordance with a previous report by Moon^84^. They found that in breast cancer, KLF4 could upregulate the expression of PFKP by directly binding to the *PFKP* promoter. More interestingly, research has shown that SP and KLF are involved in cancer metabolism, cell proliferation, apoptosis, inflammation, and tumorigenesis^85,86^. These data suggested that the transcription factors SP and KLF may be involved in the occurrence and development of cancer by PFKP, but more data are needed to support this hypothesis.

All these data suggested that promoter methylation and predicted TFs may be involved in PFKP expression and participate in pancancer metabolism, proliferation, apoptosis, inflammation, and tumorigenesis.

## Discussion

Abnormal regulation of glycolysis is a general characteristic of cancer metabolism^5,6^. It is well known that excess energy is necessary to satisfy the characteristics of excessive growth in different types of cancer. Therefore, unusual metabolic processes are regarded as a special hallmark across different cancer types. More cancer therapeutics have been developed to overcome this metabolic plasticity^87–90^. For example, 2-deoxy-glucose (2-DG), which can inhibit HK2, decreases glycolysis and induces apoptosis in cancer cells^91,92^. To provide more direction for cancer therapeutics, pancancer analysis of glycolysis reprogramming has become an important aspect.

Pancancer analysis, building on genomic, epigenomic, transcriptome, and proteomic data, aims to identify and summarize the characteristics of different cancer types^93^. However, no other pancancer analysis has focused on glycolysis. Zheng et al.^3^ found that the glycolysis-related factor SLC2A1, which plays a considerable role in cancer glycometabolism, could be explored as a potential biomarker for prognosis and immunotherapy across cancers. Their analysis indicated that a high level of SLC2A1 correlated with poor prognosis across cancers. Its function was mainly enriched in EMT, glycolysis, and the cell cycle. More importantly, there was also a remarkably positive correlation between SLC2A1 and PD-L1 or CTLA4 across cancers, which could reflect its prognosis and immunotherapy significance^94^. Moreover, Ho et al. estimated glycolysis-associated lncRNA signatures across cancers. They assessed glycolysis-associated lncRNAs with prognoses, immune infiltration, and EMT^79^. However, no additional attention has been focused on glycolytic rate-limiting enzyme pancancer analysis, which plays an important role in glycolytic processes during cancer progression.

PFKP, a well-known rate-limiting enzyme of glycolysis, plays an important role in different cancer types^95^. It also had a nonenzymatic function. For example, in T-cell acute lymphoblastic leukemia (T-ALL), PFKP stimulated T-ALL cell invasion by upregulating the expression of C-X-C chemokine receptor type 4 (CXCR4). Specifically, PFKP was shuttled into the nucleus, facilitated by Cyclin D3/CDK6. Then, nuclear PFKP enhanced the expression of CXCR4, an important chemokine receptor in T-ALL cell invasion regulation^96^. In our analysis, we also detected the subcellular location of PFKP in different cancer cells. PFKP was mainly localized in the cytoplasm, as shown by immunofluorescence data from the HPA database (Fig. S7). Additionally, immunohistochemistry of PFKP across cancers also displayed a cytoplasmic location of PFKP (Fig. 3). These data suggested that the main function of PFKP across cancers occurred in the cytoplasm and focused on its rate-limiting enzyme glycolysis.

Previous research has shown that PFKP is abnormally expressed in lung cancer, breast cancer, prostate cancer, and glioblastoma^32,33,95,97^. However, no systematic pancancer analysis of PFKP has been performed. Herein, we demonstrated that higher expression of PFKP in tumor samples was generally found across cancers.

As mentioned previously, a high level of PFKP was closely correlated with poor prognosis in cancer patients^30,98,99^. However, pancancer characteristics of PFKP have not been summarized. Our pancancer analysis data displayed that PFKP had a significant prognostic value among cancer patients. Our data also found that a high level of PFKP was related to worse OS, DSS, DFI, and PFI across cancers.

Moreover, previous data on PFKP have not displayed its diagnostic value for cancer. An accurate biomarker for cancer could help us to achieve accurate tumor prediction. Our pancancer data revealed that PFKP had a relative diagnostic accuracy in 13 cancer types, which suggested that PFKP could be a potential pancancer diagnostic biomarker. To further assess its diagnostic value, we also estimated its relationship with MSI, which is regarded as one of the important carcinogenetic factors for cancer^100,101^. However, there was no significant correlation between these factors (Fig. S8).

Recently, researchers observed a positive correlation between PFKP and PD-L1, which is the most well-known immunotherapy target. Specifically, PFKP was found to promote EGFR activation-induced PD-L1 expression by its nonmetabolic function in human GBM cells but did not display clinical relevance^66^. To assess the significance of PFKP in immunotherapy, the most promising tumor treatment, we detected the correlation between PFKP and PD-L1. A positive correlation between these factors was observed across cancers. These data suggested that PFKP plays a considerable role in tumor immunization and should be explored as an immunotherapy biomarker in the future.

Furthermore, no adequate attention was focused on the relationship between PFKP and TMB, which is also an important diagnostic and immunotherapeutic biomarker. Our study presented a positive correlation between them across cancers, which suggested that PFKP could be developed as a pancancer prognosis predictor. Overall, these data indicate that PFKP may be explored as an important prognostic predictor.

Tumor glycolysis reprogramming and immune cell infiltration are key hallmarks of cancer. Li et al.^102^ reported that high glycolytic activity was associated with immune/inflammation cell infiltration. Moreover, Tian et al.^103^ reported that in osteosarcoma, glycolysis-immune-related genes could predict patient prognosis. However, no correlation between glycolysis-related kinase and immune cell infiltration has been reported. Our data suggested that the glycolysis-related kinase PFKP was highly expressed across cancers and positively correlated with tumor immune cell infiltration and immune-related genes, suggesting that PFKP could be an important immunoregulation marker.

Previous data revealed that the function of PFKP was mainly focused on its glycolytic regulation. For example, PFKP is highly expressed in lung cancer and regulates cell proliferation by regulating glycolytic activity^29^. Moreover, PFKP also regulates glucose starvation-induced metabolic stress in lung cancer cells by fatty acid oxidation in an AMPK-ACC2-dependent manner^95^. In glioma stem cells (GSCs), PFKP regulates metabolism and phenotypic reprogramming by interacting with mitochondrial membrane protein voltage-dependent anion channel 2 (VDAC2)^104^. Moreover, PFKP could also be involved in cell invasion and metastasis in breast cancer and oral squamous cell carcinoma^33,105^. Our data suggested that the function of PFKP was mainly enriched in Wnt, EGFR, and PI3K signaling, which are important cell proliferation pathways^106–111^.

Moreover, our functional enrichment analysis displayed that the DEGs of PFKP were mainly enriched in the G2/M checkpoint, and no previous data have reported this phenomenon. Our pancancer GSEA also revealed a significant positive correlation between PFKP and EMT, suggesting that PFKP may also be involved in tumor metastasis, which was in accordance with previous research in breast cancer and T-cell acute lymphoblastic leukemia^33,96^. GSEA data also indicated that PFKP could participate in tumor immune regulation because of its close connection with the inflammatory response and TNF-α signaling via NF-κB hallmarks.

Furthermore, drug resistance is a major challenge in cancer treatment^112^. No additional analysis estimated the effect of PFKP on cancer drug resistance. Our data implied that patients with higher levels of PFKP had a lower sensitivity to several cancer drugs. These data suggested a fine drug sensitivity of PFKP. However, the intrinsic mechanism of this relationship requires further analysis.

Finally, upstream expression regulation of PFKP was also assessed by promoter DNA methylation and transcription factor prediction. First, our data found that promoter DNA methylation may play a partial regulatory role in high levels of PFKP across many cancer types. This finding needs more supporting experimental data. Then, we also analyzed the promoter sequence of PFKP, and several transcription factors with higher enrichment scores were predicted, such as the KLF transcription factor family and SP family, which were also reported by other researchers^84,85^.

In summary, our data revealed that PFKP was highly expressed in most cancer types and was strongly correlated with poor patient prognosis. Moreover, PFKP may be a useful clinical diagnostic marker due to its positive correlation with TMB and PD-L1. Additionally, PFKP could also be a predictive immunoregulation marker and drug sensitivity indicator. Finally, the functions of PFKP were mainly enriched in cell cycle operation and tumor metastasis. The expression of PFKP may be regulated by DNA methylation and the transcription factors KLF and SP.

### Supplementary Information

Supplementary Figure S1.

Supplementary Figure S2.

Supplementary Figure S3.

Supplementary Figure S4.

Supplementary Figure S5.

Supplementary Figure S6.

Supplementary Figure S7.

Supplementary Figure S8.

Supplementary Legends.

Supplementary Table S1.

## Data Availability

The datasets during and/or analyzed during the current study are available from the corresponding author upon reasonable request.

## Abbreviations

2-DG: 2-deoxy-glucose
ACC: Adrenocortical carcinoma
ATP: Adenosine triphosphate
AUC: Area under the curve
BLCA: Bladder urothelial carcinoma
BRCA: Breast invasive carcinoma
CCLE: Cancer Cell Line Encyclopedia
CESC: Cervical squamous cell carcinoma and endocervical adenocarcinoma
CHOL: Cholangiocarcinoma
COAD: Colon adenocarcinoma
CTLA4: Cytotoxic T-lymphocyte associated protein 4
CXCR4: C-X-C chemokine receptor type 4
DEGs: Differentially expression genes
DFI: Disease free interval
DLBC: Lymphoid neoplasm diffuse large B-cell Lymphoma
DSS: Disease-specific survival
EMT: Epithelial mesenchymal transition
ESCA: Esophageal carcinoma
GBM: Glioblastoma multiforme
GSCs: Glioma stem cells
GTEx: Genotype-Tissue Expression Project
HER2: Human epidermal growth factor receptor 2
HK: Hexokinase
HNSC: Head and Neck squamous cell carcinoma
HPA: Human protein atlas
KICH: Kidney chromophobe
KIRC: Kidney renal clear cell carcinoma
KIRP: Kidney renal papillary cell carcinoma
KLF: Kruppel family of transcription factors
LAML: Acute myeloid leukemia
LGG: Brain lower grade glioma
LIHC: Liver hepatocellular carcinoma
LUAD: Lung adenocarcinoma
LUSC: Lung squamous cell carcinoma
MESO: Mesothelioma
OS: Overall survival
OV: Ovarian serous cystadenocarcinoma
PAAD: Pancreatic adenocarcinoma
pAb: Polyclonal antibody
PCPG: Pheochromocytoma and paraganglioma
PD-L1: Programmed cell death-Ligand 1
PFI: Progression-free interval
PFK-1: Phosphofructokinase 1
PFKL: Phosphofructokinase, liver type
PFKM: Phosphofructokinase, muscle
PFKP: Phosphofructokinase, platelet
PK: Pyruvate kinase
PRAD: Prostate adenocarcinoma
READ: Rectum adenocarcinoma
ROC: Receiver operating characteristic
SARC: Sarcoma
SKCM: Skin cutaneous melanoma
STAD: Stomach adenocarcinoma
TCGA: The cancer genome atlas
TEC: T-cell development kinases
TFs: Transcription factors
TGCT: Testicular germ cell tumors
THCA: Thyroid carcinoma
THYM: Thymoma
TMB: Tumor mutational burden
UCEC: Uterine corpus endometrial carcinoma
UCS: Uterine carcinosarcoma
UVM: Uveal melanoma
VDAC2: Voltage-dependent anion channel 2
