# Supplementary figures and images for: PFKP is a prospective prognostic, diagnostic, immunological and drug sensitivity predictor across pan-cancer
Source: Sci Rep. 2023 Oct 13;13:17399. doi: 10.1038/s41598-023-43982-2 (PMC10576092; doi:10.1038/s41598-023-43982-2)

**A**PFKP expression ( $\text{Log}_2(\text{TPM}+1)$ )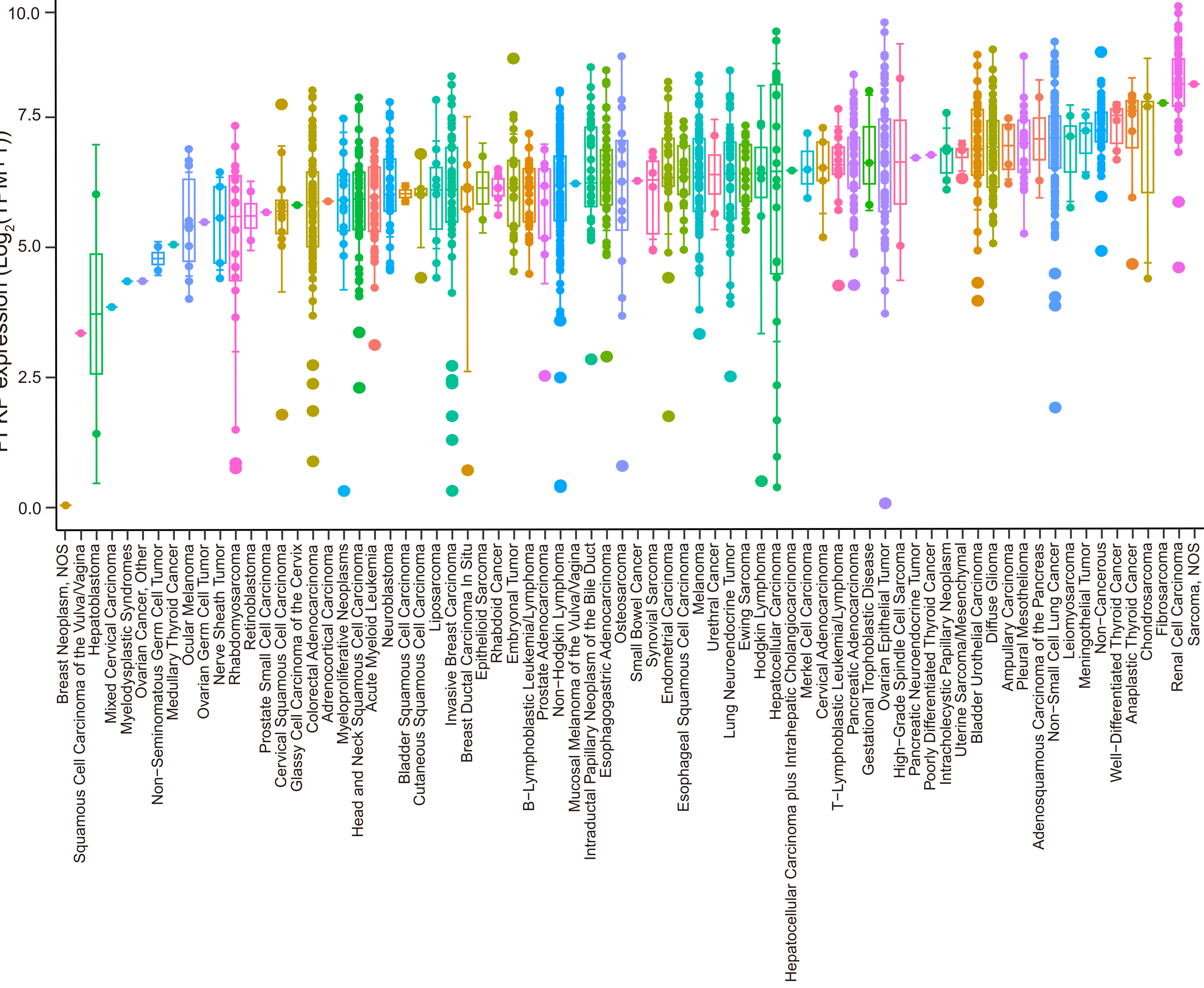**B**

PFKP expression in TCGA tumor samples

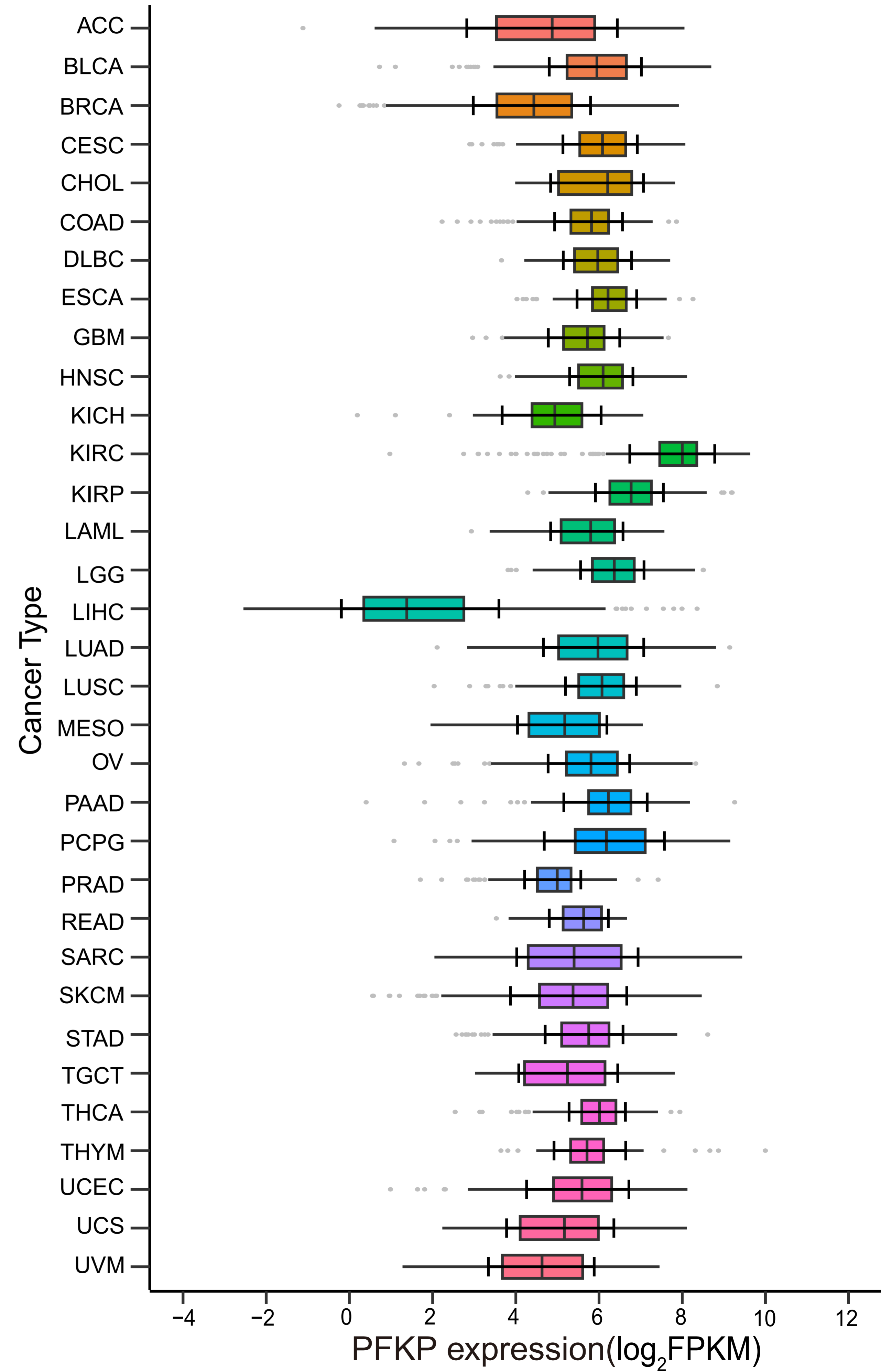

Supplement: Supplementary file 1 — Supplementary Figure S1. [file 41598_2023_43982_MOESM1_ESM.pdf]

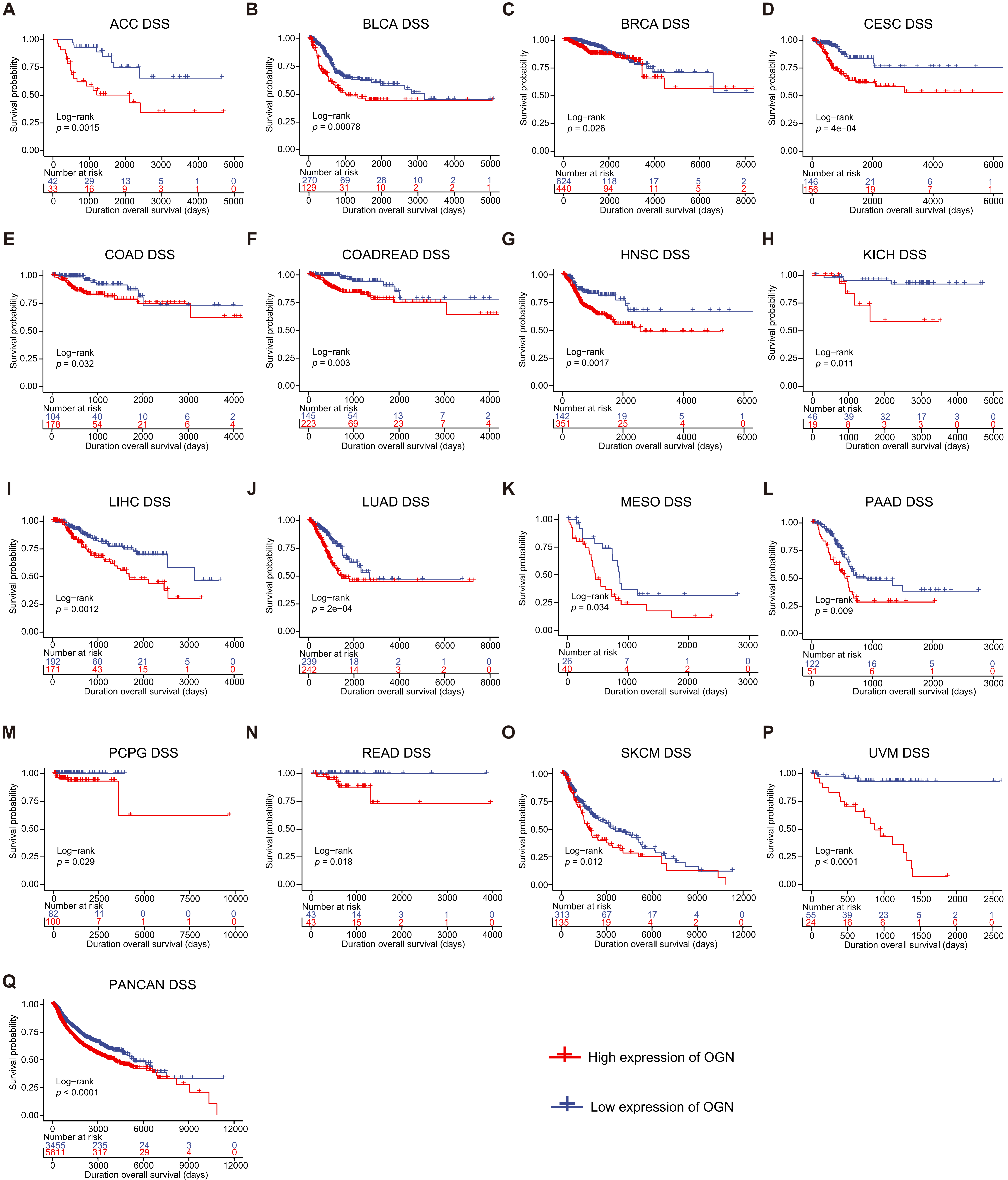

Supplement: Supplementary file 2 — Supplementary Figure S2. [file 41598_2023_43982_MOESM2_ESM.pdf]

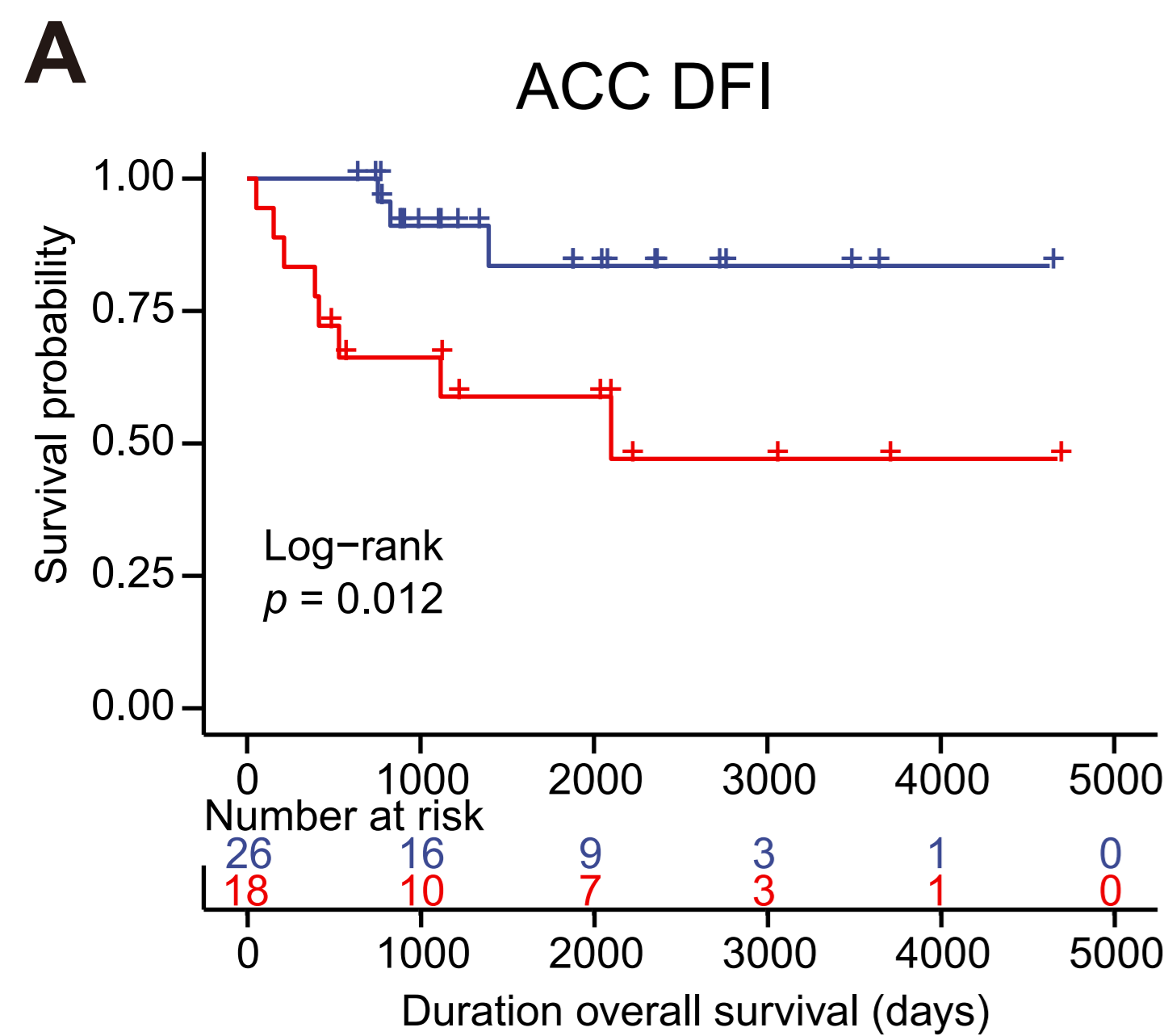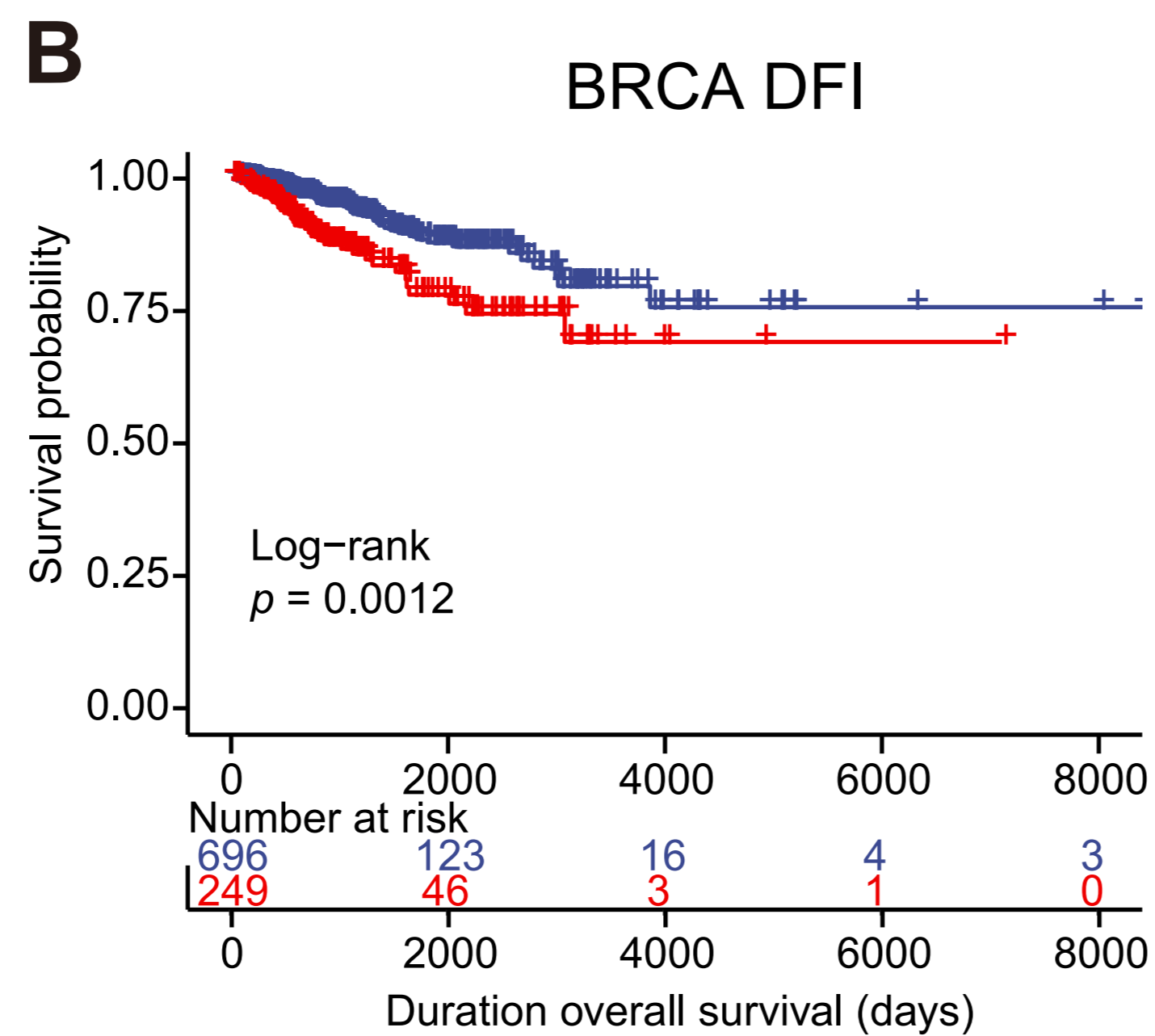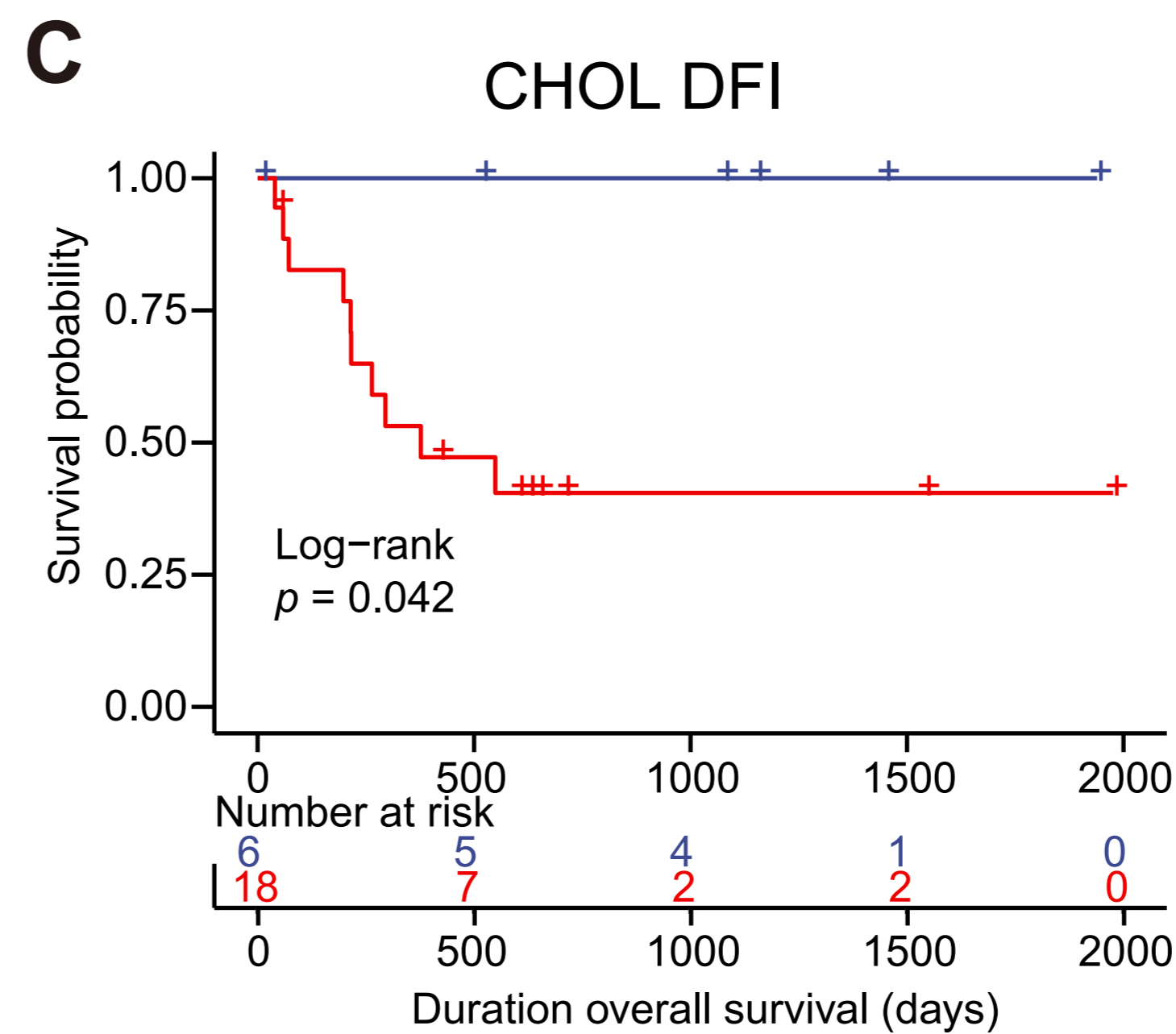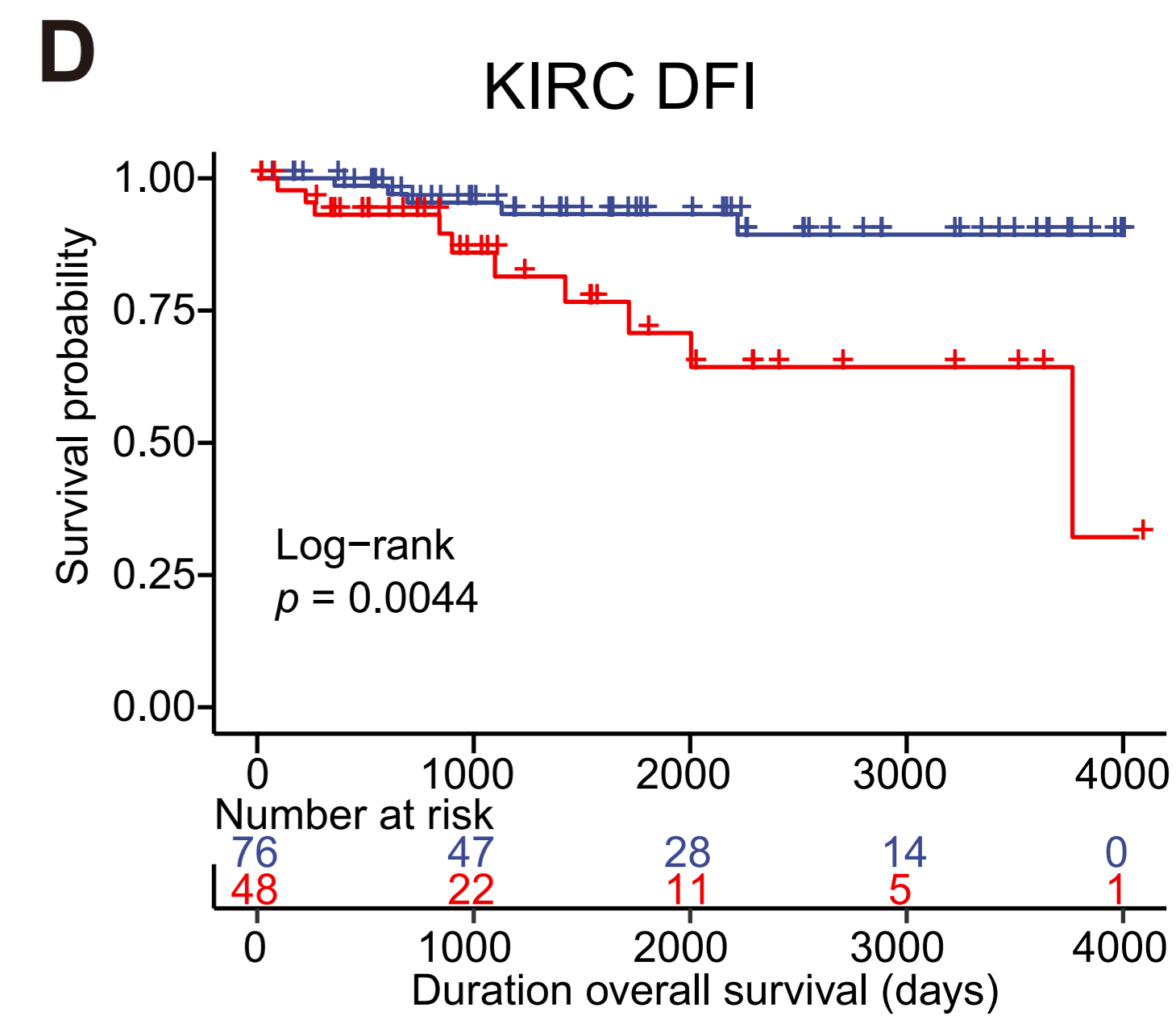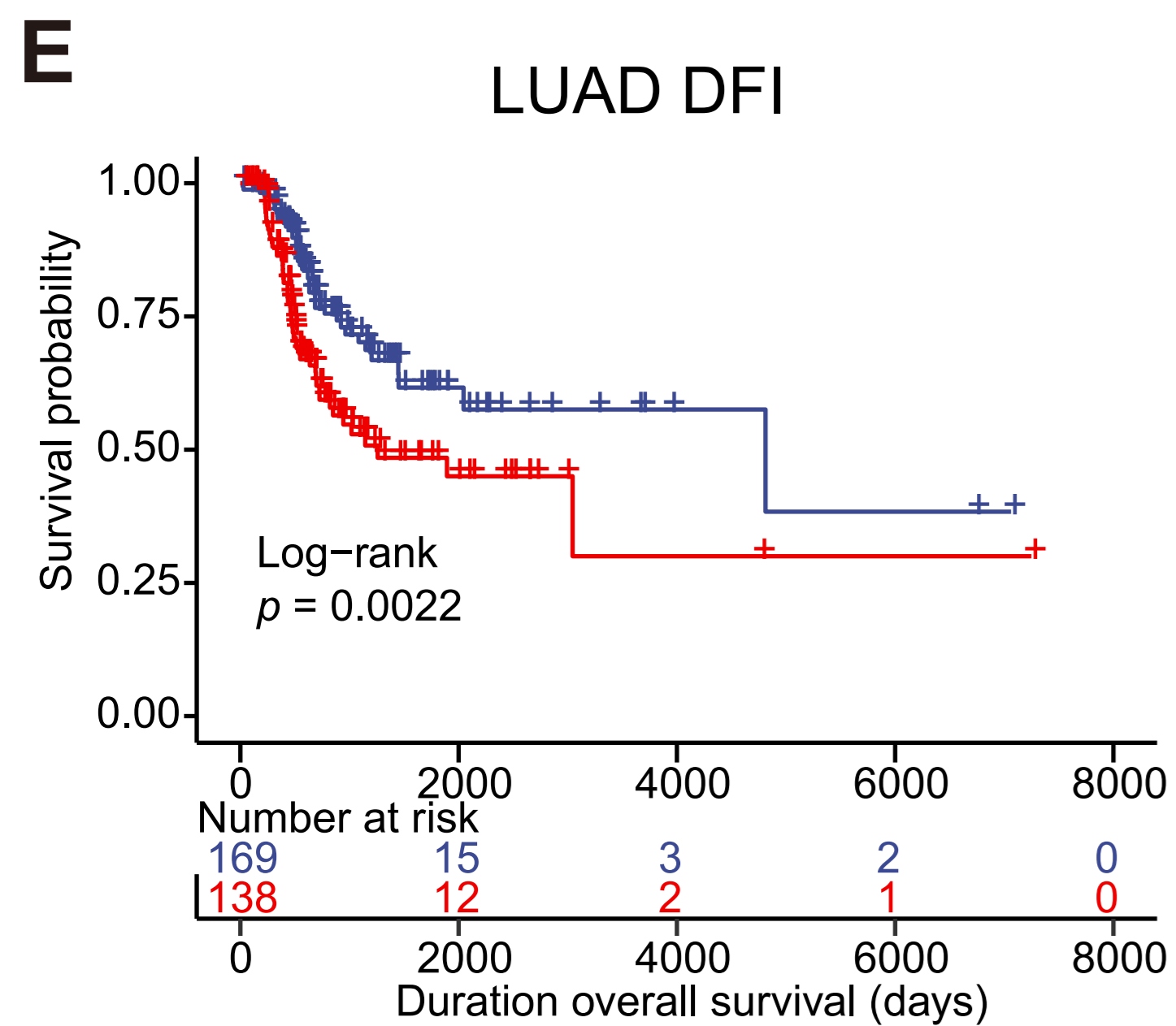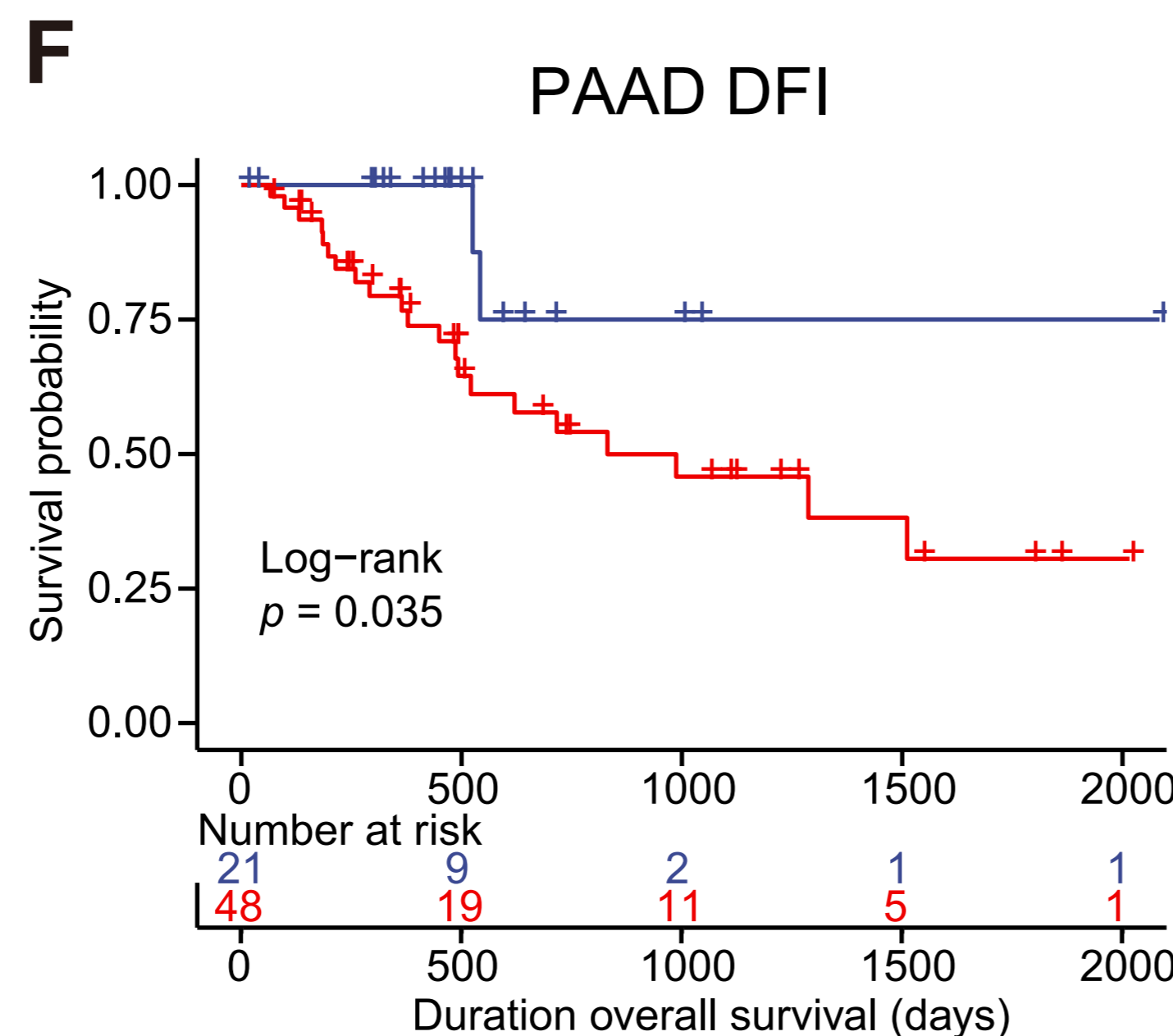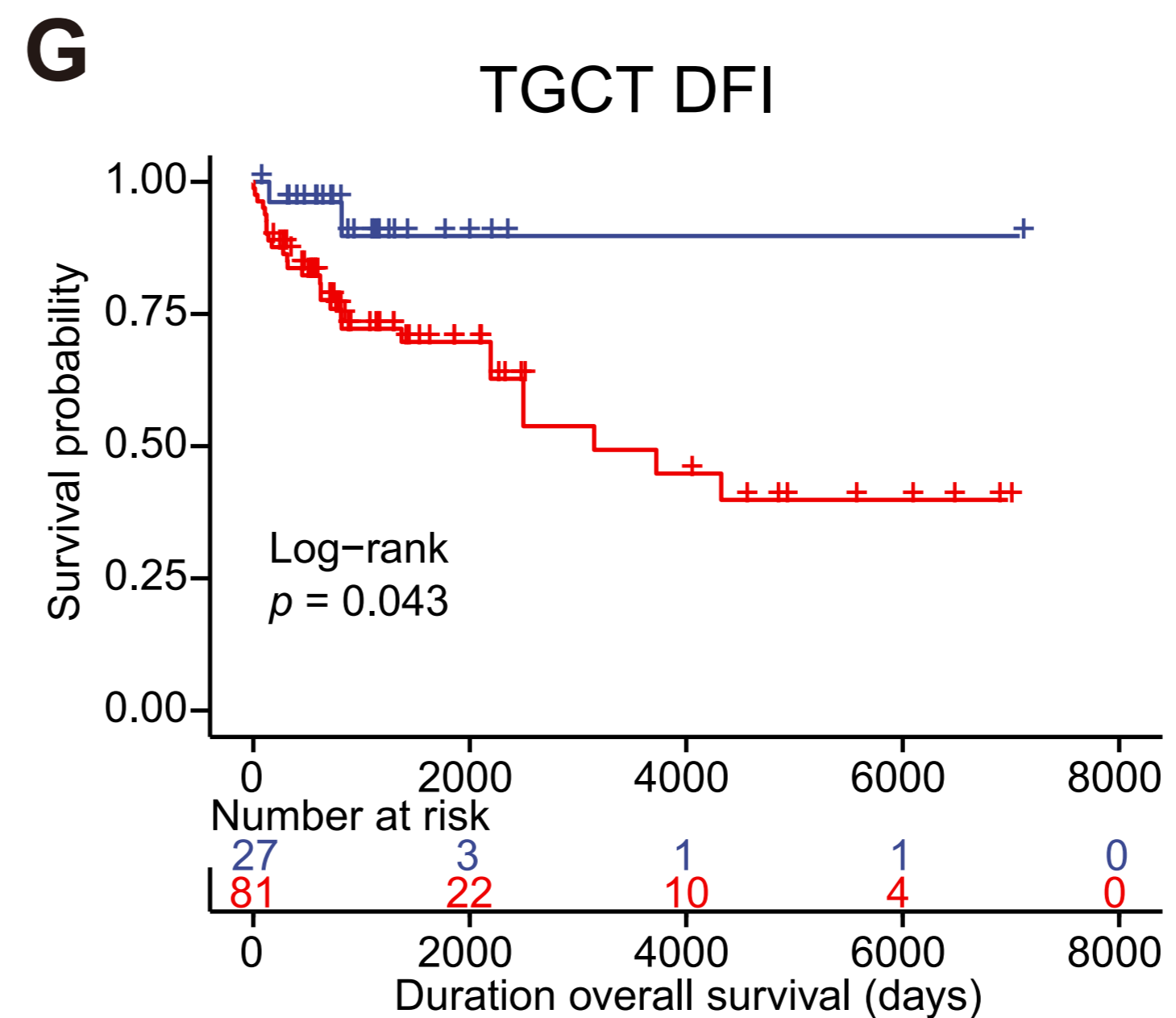

—+— High expression of OGN

—+— Low expression of OGN

Supplement: Supplementary file 3 — Supplementary Figure S3. [file 41598_2023_43982_MOESM3_ESM.pdf]

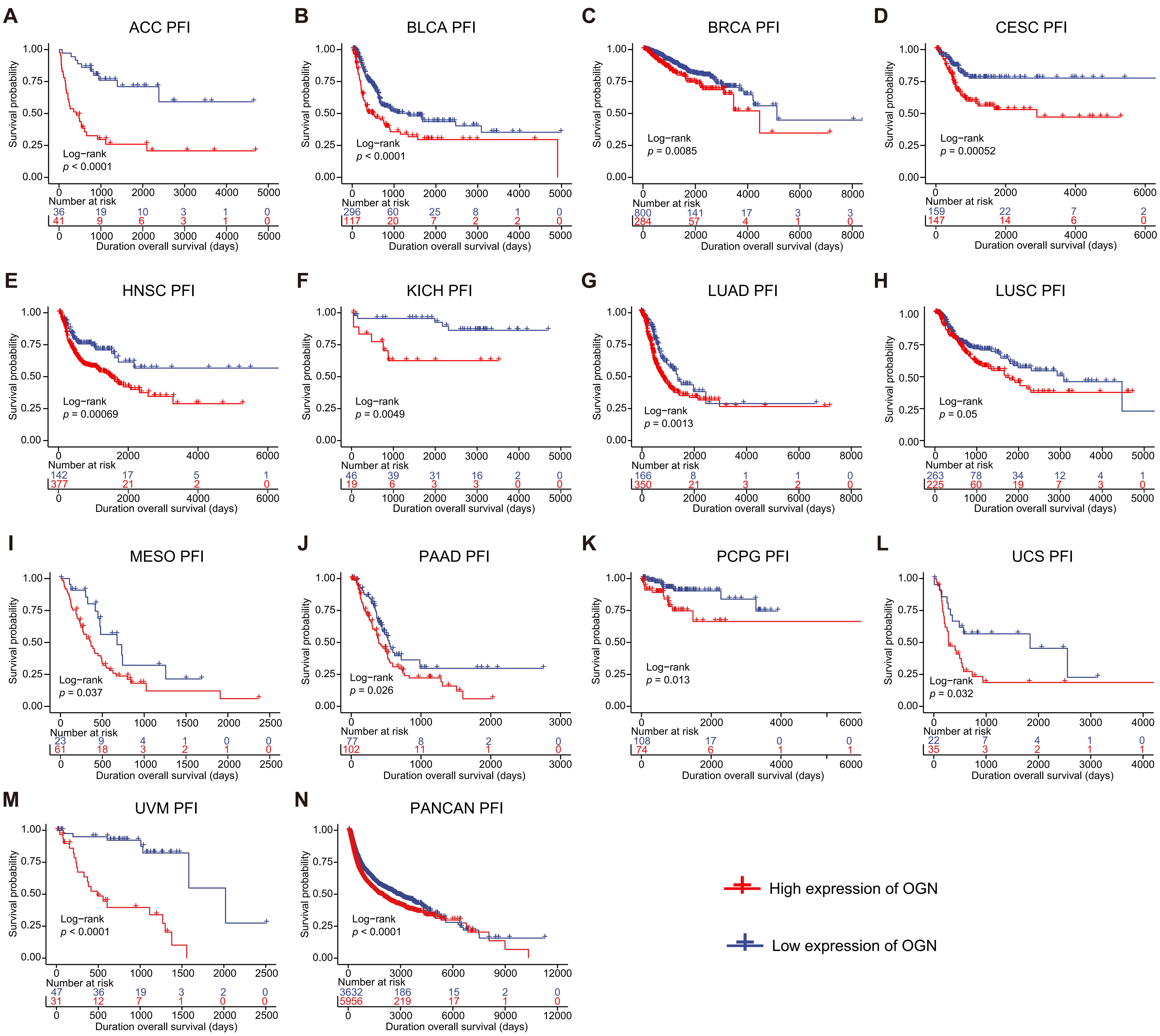

Supplement: Supplementary file 4 — Supplementary Figure S4. [file 41598_2023_43982_MOESM4_ESM.pdf]

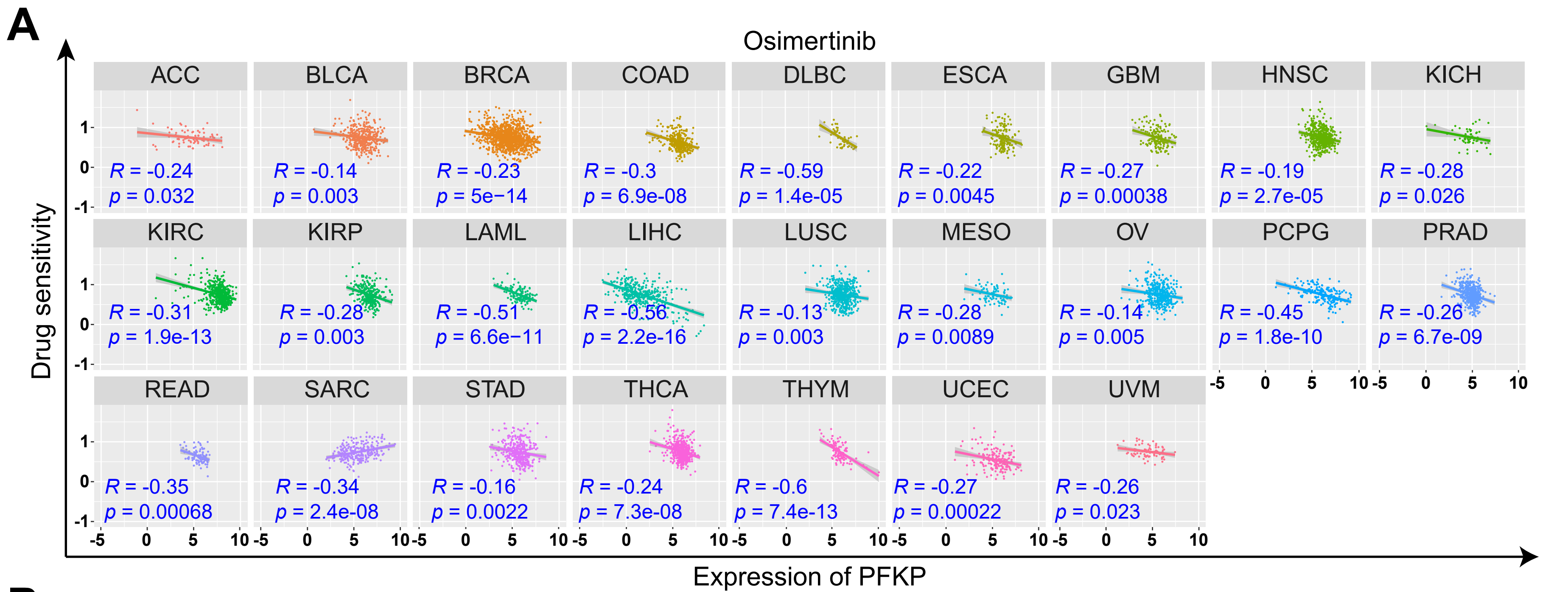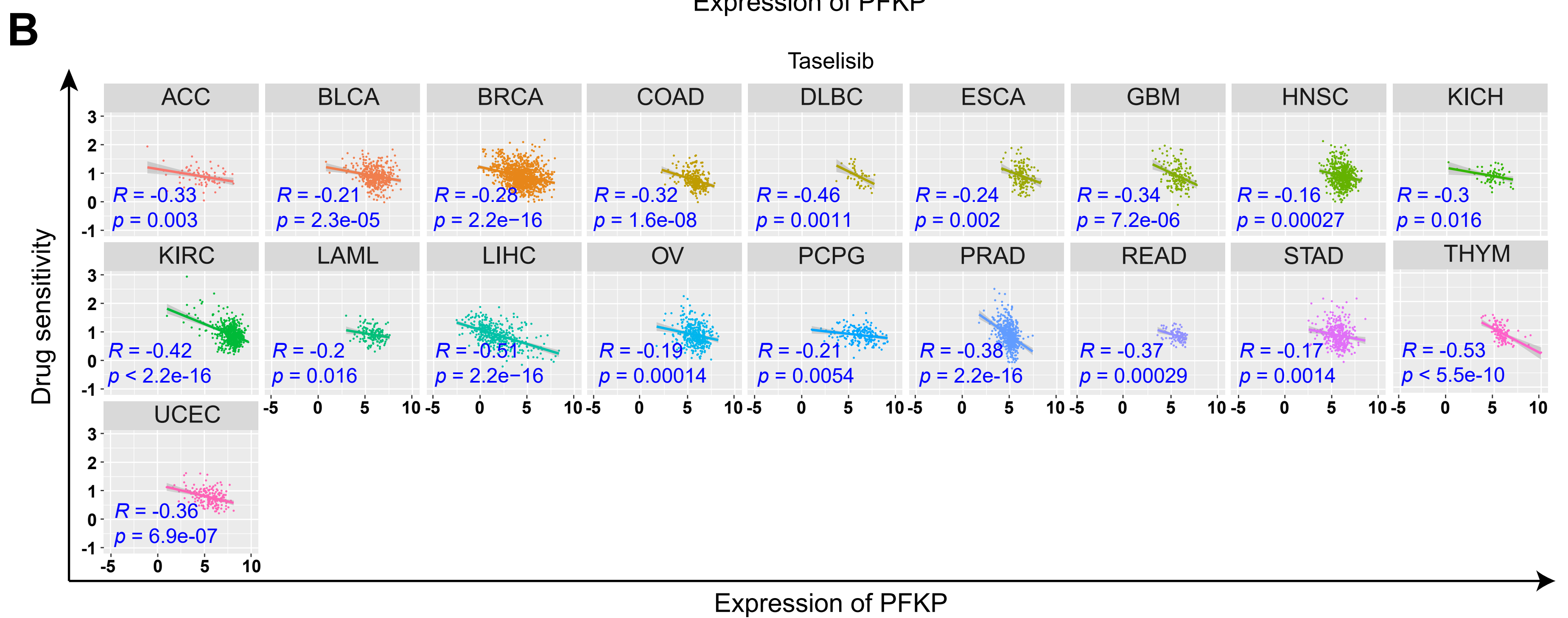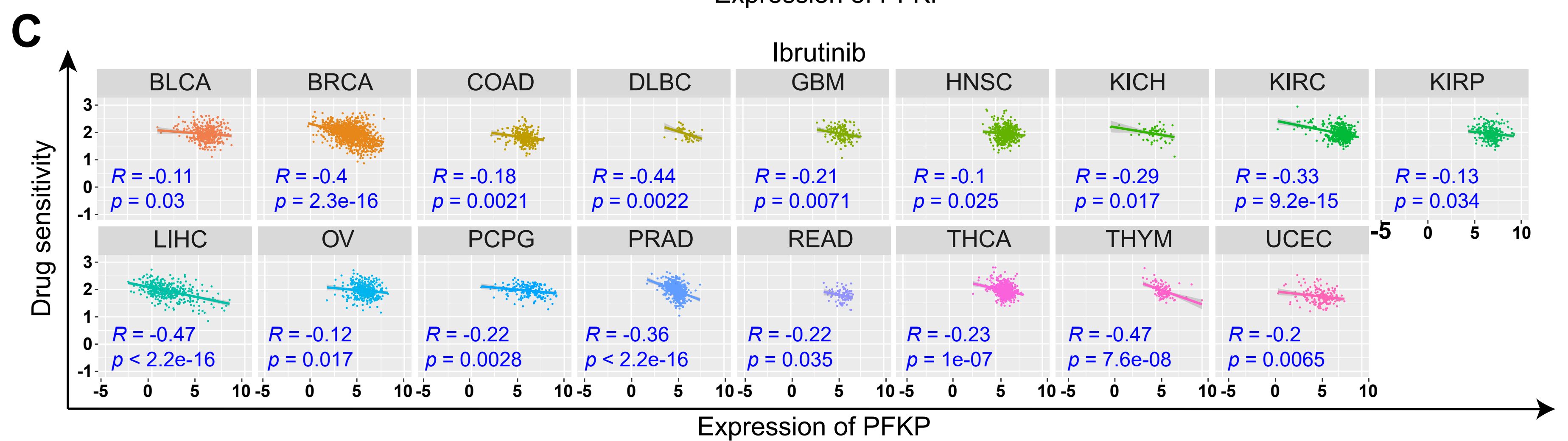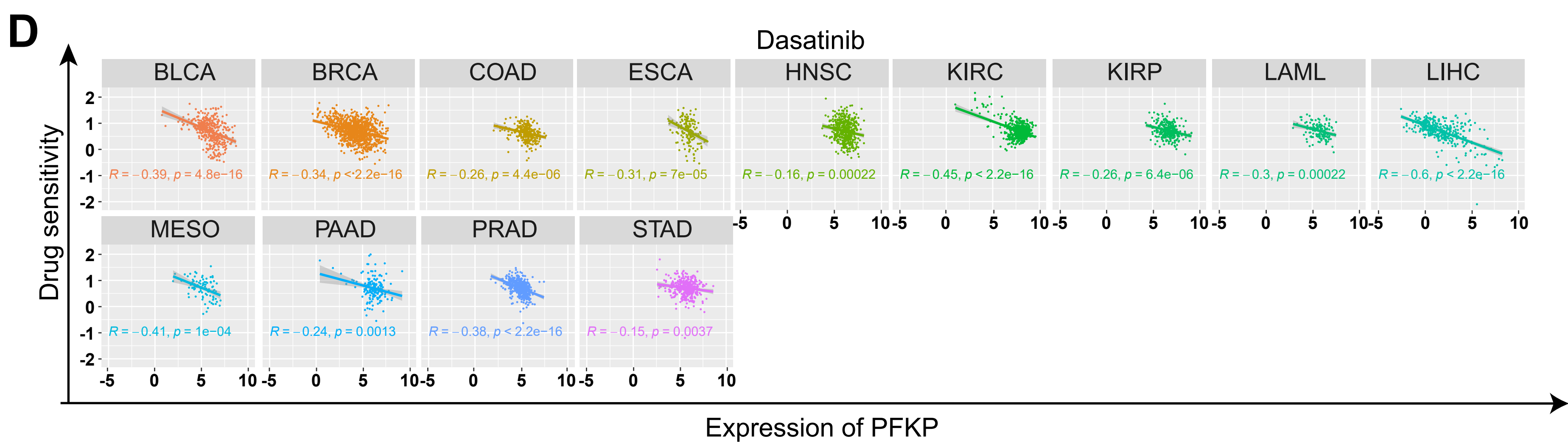

Supplement: Supplementary file 6 — Supplementary Figure S6. [file 41598_2023_43982_MOESM6_ESM.pdf]

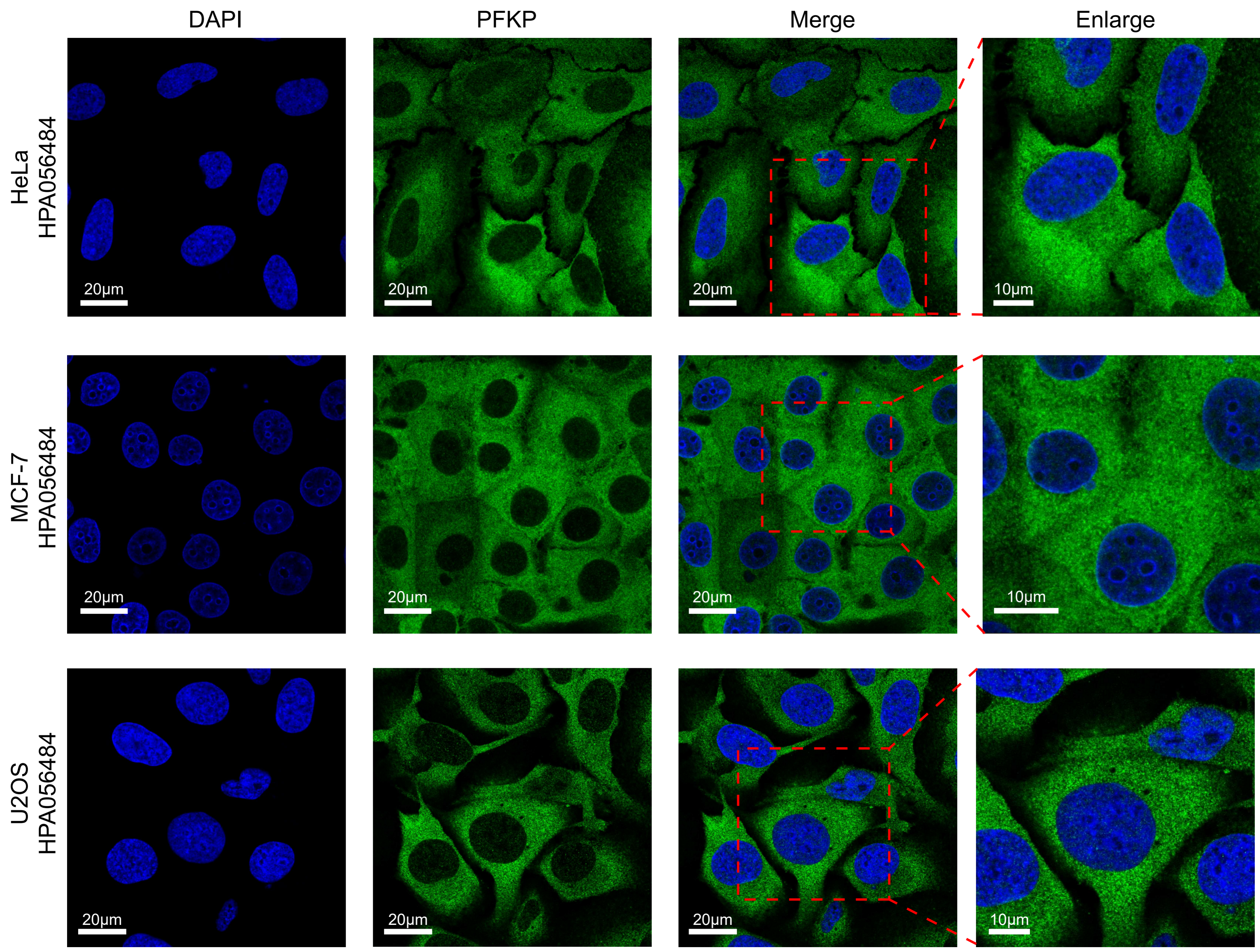

Supplement: Supplementary file 7 — Supplementary Figure S7. [file 41598_2023_43982_MOESM7_ESM.pdf]

Correlation between PFKP and Microsatellite instability(MSI)

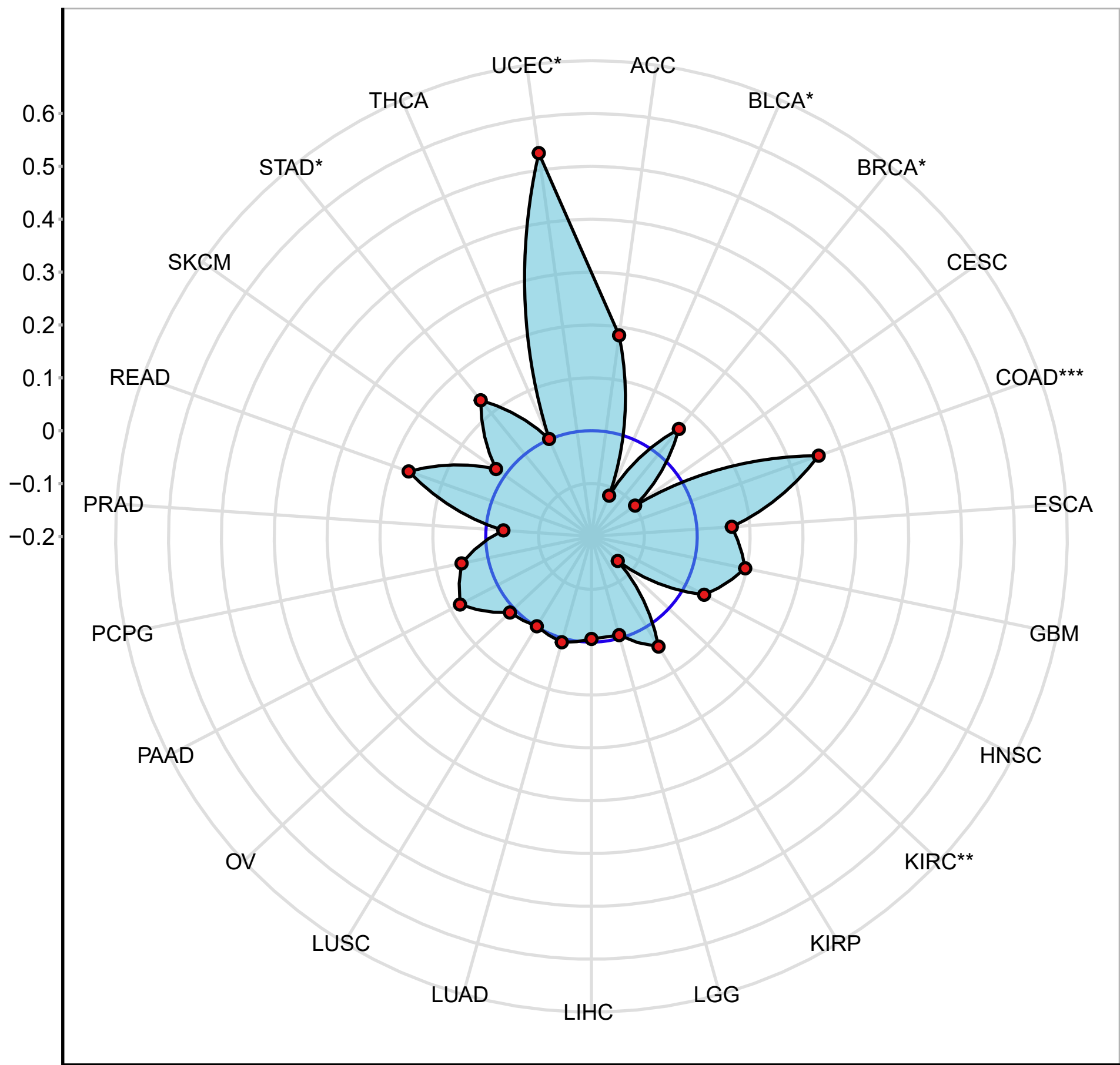

Supplement: Supplementary file 8 — Supplementary Figure S8. [file 41598_2023_43982_MOESM8_ESM.pdf]
