# Supplementary material for: PFKP is a prospective prognostic, diagnostic, immunological and drug sensitivity predictor across pan-cancer
Source: Sci Rep. 2023 Oct 13;13:17399. doi: 10.1038/s41598-023-43982-2 (PMC10576092; doi:10.1038/s41598-023-43982-2)
Supplement: Supplementary file 5 — Supplementary Figure S5. [file 41598_2023_43982_MOESM5_ESM.pdf]

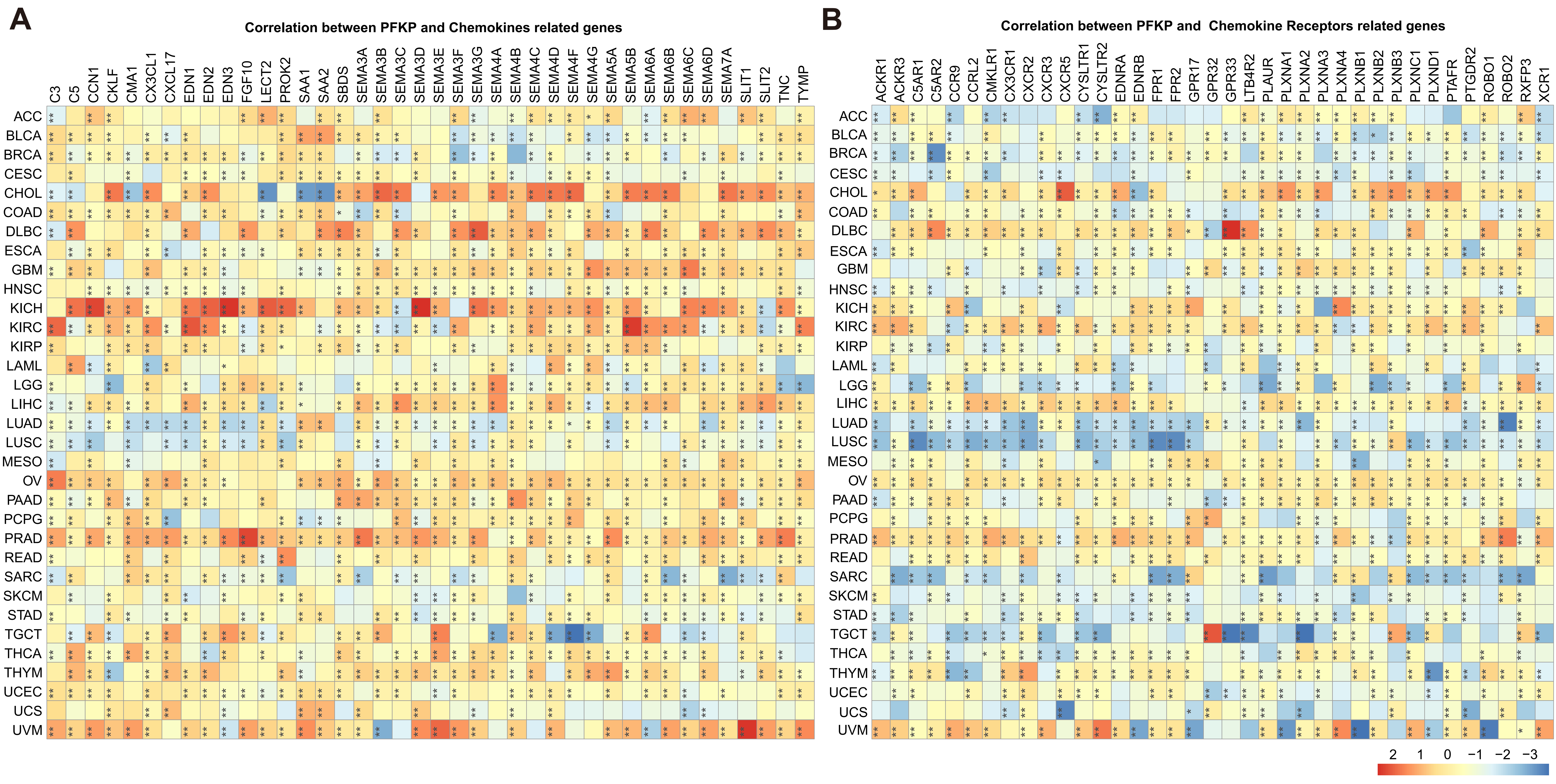
A
Correlation between PFKP and Chemokines related genes
C3
C5
CCN1
CKLF
CMA1
CX3CL1
CXCL17
EDN1
EDN2
EDN3
FGF10
LECT2
PROK2
SAA1
SAA2
SBDS
SEMA3A
SEMA3B
SEMA3C
SEMA3D
SEMA3E
SEMA3F
SEMA3G
SEMA4A
SEMA4B
SEMA4C
SEMA4D
SEMA4F
SEMA4G
SEMA5A
SEMA5B
SEMA6A
SEMA6B
SEMA6C
SEMA6D
SEMA7A
SLIT1
SLIT2
TNC
TYMP
ACC
BLCA
BRCA
CESC
CHOL
COAD
DLBC
ESCA
GBM
HNSC
KICH
KIRC
KIRP
LAML
LGG
LIHC
LUAD
LUSC
MESO
OV
PAAD
PCPG
PRAD
READ
SARC
SKCM
STAD
TGCT
THCA
THYM
UCEC
UCS
UVM
B
Correlation between PFKP and Chemokine Receptors related genes
ACKR1
ACKR3
C5AR1
C5AR2
CCR9
CCRL2
CMKLR1
CX3CR1
CXCR2
CXCR3
CXCR5
CYSLTR1
CYSLTR2
EDNRA
EDNRB
FPR1
FPR2
GPR17
GPR32
GPR33
LTB4R2
PLAUR
PLXNA1
PLXNA2
PLXNA3
PLXNA4
PLXNB1
PLXNB2
PLXNB3
PLXNC1
PLXND1
PTAFR
PTGDR2
ROBO1
ROBO2
RXFP3
XCR1
ACC
BLCA
BRCA
CESC
CHOL
COAD
DLBC
ESCA
GBM
HNSC
KICH
KIRC
KIRP
LAML
LGG
LIHC
LUAD
LUSC
MESO
OV
PAAD
PCPG
PRAD
READ
SARC
SKCM
STAD
TGCT
THCA
THYM
UCEC
UCS
UVM
2
1
0
−1
−2
−3
