# Supplementary material for: PFKP is a prospective prognostic, diagnostic, immunological and drug sensitivity predictor across pan-cancer
Source: Sci Rep. 2023 Oct 13;13:17399. doi: 10.1038/s41598-023-43982-2 (PMC10576092; doi:10.1038/s41598-023-43982-2)
Supplement: Supplementary file 9 — Supplementary Legends. [file 41598_2023_43982_MOESM9_ESM.docx]

**Supplementary figures legend**

**Figure S1. Differential expression of PFKP in cancer cell lines and tumor tissue samples were analyzed in CCLE and TCGA, respectively. (A)** Expression of PFKP in different cancer cell lines were accessed from CCLE. TPM, Transcripts Per Kilobase Million. **(B)** Expression of PFKP in tumor samples were showed across 33 cancer types. TPM, Transcript per Kilobase per Million mapped reads. FPKM, fragments per kilobase million.

**Figure S2. Effect of PFKP expression on** **disease specific survival (DSS) were performed pan-cancer.** Disease specific survival analysis of PFKP were accessed by Log-Rank test survival analysis in ACC **(A)**, BLCA **(B)**, BRCA **(C)**, CESC **(D)**, COAD **(E)**, COADREAD **(F)**, HNSC **(G)**, KICH **(H)**, LIHC **(I)**, LUAD **(J)**, MESO **(K)**, PAAD **(L)**, PCPG **(M)**, READ **(N),** SKCM **(O),** UVM **(P)** and overall cancer patients (PANCAN) **(Q)**.

**Figure S3. Effect of PFKP expression on disease free interval (DFI) were performed pan-cancer.** Disease free interval analysis of PFKP were accessed by Log-Rank test survival analysis in ACC **(A)**, BRCA **(B)**, CHOL **(C)**, KIRC **(D)**, LUAD **(E)**, PAAD **(F)**, and TGCT **(G)**.

**Figure S4. Effect of PFKP expression on progression free interval (PFI) were performed pan-cancer.** Progression free interval analysis of PFKP were accessed by Log-Rank test survival analysis in ACC **(A)**, BLCA **(B)**, BRCA **(C)**, CESC **(D)**, HNSC **(E)**, KICH **(F)**, LUAD **(G)**, LUSC **(H)**, MESO **(I)**, PAAD **(J),** PCPG **(K),** USC **(L)**, UVM **(M)**, and overall cancer patients (PANCAN) **(N)**.

**Figure S5. Pearson correlation analysis between PFKP and immune related genes across pan-cancer were displayed by heatmap.** Pearson correlation analysis between PFKP and chemokines related genes **(A)** or chemokine receptors related genes **(B)** were displayed by heatmaps.

**Figure S6. Effect of PFKP on the sensitivity of Osimertinib (A), Taselisib (B), Ibrutinib (C), and Dasatinib (D) were displayed by scatter plots.**

**Figure S7. Subcellular location of PFKP was analyzed by immunofluorescence in HeLa, MCF-7, and U2OS cells from HPA.** DAPI, nuclear marker. HPA056484, antibody id of PFKP in HPA. Scale bar, 20μm and 10μm.

**Figure S8. Correlation between PFKP and microsatellite instability (MSl) across pan-cancer were displayed by radar chart.**  **p* < 0.05, ***p* < 0.01, ****p* < 0.001, *****p* < 0.0001.
