# Supplementary material for: PFKP is a prospective prognostic, diagnostic, immunological and drug sensitivity predictor across pan-cancer
Source: Sci Rep. 2023 Oct 13;13:17399. doi: 10.1038/s41598-023-43982-2 (PMC10576092; doi:10.1038/s41598-023-43982-2)
Supplement: Supplementary file 10 — Supplementary Table S1. [file 41598_2023_43982_MOESM10_ESM.docx]

**Table S1**

JASPAR analysis results for top 20 transcription factor binding sites within the promoter of PFKP gene (Sequence ID: GRCh38: NC_000010.11:3065548-3067548).

| TF | Item | | Score | Position | Genomic Size | Strand |
| --- | --- | --- | --- | --- | --- | --- |
| KLF15 | MA1513.1 | | 614 | chr10:3066821-3066831 | 11 | - |
| POU4F1 | MA0790.1 | | 610 | chr10:3066041-3066054 | 14 | + |
| EWSR1-FLI1 | MA0149.1 | | 605 | chr10:3066778-3066795 | 18 | - |
| ZNF320 | MA1976.1 | | 604 | chr10:3067055-3067081 | 27 | - |
| SP5 | MA1965.1 | | 602 | chr10:3067424-3067433 | 10 | + |
| PATZ1 | MA1961.1 | | 580 | chr10:3066820-3066831 | 12 | + |
| POU4F3 | MA0791.1 | | 576 | chr10:3066041-3066056 | 16 | + |
| KLF4 | MA0039.4 | | 573 | chr10:3065595-3065606 | 12 | + |
| EGR1 | MA0162.4 | | 571 | chr10:3067490-3067503 | 14 | - |
| TRPS1 | MA1970.1 | | 569 | chr10:3066209-3066220 | 12 | + |
| KLF5 | | MA0599.1 | 554 | chr10:3066666-3066675 | 10 | - |
| ZNF148 | | MA1653.1 | 553 | chr10:3066992-3067003 | 12 | + |
| PRDM9 | | MA1723.1 | 549 | chr10:3066511-3066534 | 24 | - |
| ZNF281 | | MA1630.2 | 546 | chr10:3066821-3066832 | 12 | + |
| CTCFL | | MA1102.2 | 544 | chr10:3067144-3067155 | 12 | - |
| SP1 | | MA0079.5 | 541 | chr10:3066822-3066830 | 9 | + |
| KLF1 | | MA0493.2 | 541 | chr10:3066822-3066830 | 9 | + |
| SP2 | | MA0516.3 | 541 | chr10:3066822-3066830 | 9 | + |
| SP4 | | MA0685.2 | 541 | chr10:3066822-3066830 | 9 | + |
| KLF14 | | MA0740.2 | 541 | chr10:3066822-3066830 | 9 | + |
| KLF12 | | MA0742.2 | 541 | chr10:3066822-3066830 | 9 | + |
| KLF10 | | MA1511.2 | 541 | chr10:3066822-3066830 | 9 | + |
| KLF7 | | MA1959.1 | 541 | chr10:3066822-3066830 | 9 | + |
| MAZ | | MA1522.1 | 539 | chr10:3066992-3067002 | 11 | + |
| TFAP2B | | MA0811.1 | 536 | chr10:3066128-3066139 | 12 | - |
